# Exocyst components promote an incompatible interaction between *Glycine max* (soybean) and *Heterodera glycines* (the soybean cyst nematode)

**DOI:** 10.1038/s41598-020-72126-z

**Published:** 2020-09-14

**Authors:** Keshav Sharma, Prakash M. Niraula, Hallie A. Troell, Mandeep Adhikari, Hamdan Ali Alshehri, Nadim W. Alkharouf, Kathy S. Lawrence, Vincent P. Klink

**Affiliations:** 1 Department of Biological Sciences, Mississippi State University, Mississippi State, MS 39762 USA; 2 Department of Mathematics and Computer Science, Texas Women’s University, Denton, TX 76204 USA; 3 Department of Computer and Information Sciences, Towson University, Towson, MD 21252 USA; 4 Department of Entomology and Plant Pathology, Auburn University, 209 Life Science Building, Auburn, AL 36849 USA; 5 Department of Biochemistry, Molecular Biology, Entomology and Plant Pathology, Mississippi State University, Mississippi State, MS 39762 USA; 6 Center for Computational Sciences High Performance Computing Collaboratory, Mississippi State University, Mississippi State, MS 39762 USA; 7 Present Address: USDA-ARS Cereal Disease Laboratory, University of Minnesota, 1551 Lindig Street, St. Paul, MN 55108 USA; 8 Present Address: Department of Plant Pathology and Microbiology, Texas A&M AgriLife Research and Extension Center, Texas A&M University, 2415 E. Hwy. 83, Weslaco, TX 78596 USA

**Keywords:** Plant cell biology, Plant genetics, Plant immunity, Plant molecular biology, Cell biology, Plant sciences

## Abstract

Vesicle and target membrane fusion involves tethering, docking and fusion. The GTPase *SECRETORY4* (*SEC4*) positions the exocyst complex during vesicle membrane tethering, facilitating docking and fusion. *Glycine max* (soybean) Sec4 functions in the root during its defense against the parasitic nematode *Heterodera glycines* as it attempts to develop a multinucleate nurse cell (syncytium) serving to nourish the nematode over its 30-day life cycle. Results indicate that other tethering proteins are also important for defense. The *G. max* exocyst is encoded by 61 genes: 5 EXOC1 (Sec3), 2 EXOC2 (Sec5), 5 EXOC3 (Sec6), 2 EXOC4 (Sec8), 2 EXOC5 (Sec10) 6 EXOC6 (Sec15), 31 EXOC7 (Exo70) and 8 EXOC8 (Exo84) genes. At least one member of each gene family is expressed within the syncytium during the defense response. Syncytium-expressed exocyst genes function in defense while some are under transcriptional regulation by mitogen-activated protein kinases (MAPKs). The exocyst component EXOC7-H4-1 is not expressed within the syncytium but functions in defense and is under MAPK regulation. The tethering stage of vesicle transport has been demonstrated to play an important role in defense in the *G. max*-*H. glycines* pathosystem, with some of the spatially and temporally regulated exocyst components under transcriptional control by MAPKs.

## Introduction

During their defense against pathogen infection, plants employ cellular processes to detect and amplify signals derived from the activities of those pathogens. If successful, these plant processes lead to resistance^1^. Among the better known defense processes that lead to resistance are those mediated by resistance (R) proteins that function as pattern recognition receptors (PRRs)^1^. PRRs themselves function within the context of effector-triggered immunity (ETI) and pathogen-associated molecular pattern-triggered immunity (PTI) processes^1–5^. Additionally, among these defense proteins is a cellular apparatus that functions in vesicle transport^6,7^. Upon its secretion, this vesicle transport apparatus delivers cargo that inhibits pathogen infection^6,7^. The apparatus also delivers and internalizes PRRs, facilitating defense^6,7^.

Through a genetic approach, Novick et al*.*^8^ analyzed the vesicle transport apparatus using the ascomycete fungus *Saccharomyces cerevisiae*. In generating secretory (*sec*) mutants, Novick et al*.*^8^ identified genes whose protein products are responsible for vesicle transport. Unrelated experiments using the plant genetic model *Arabidopsis thaliana* have aided in the identification of PENETRATION1 (PEN1)^6,9–11^. PEN1 is a syntaxin and the first protein among plant vesicle transport protein homologs shown to function in defense^6,11^. Consequently, this work revealed that vesicle transport proteins are universal among eukaryotes and important to the defense process^9,10^.

Syntaxin functions as part of a membrane receptor complex known as soluble N-ethylmaleimide-sensitive fusion protein attachment protein receptor (SNARE). SNARE itself is part of an even larger complex known as the 20 S particle. The 20 S particle was identified through work on *Rattus norvegicus* (rat) liver tissue using purified recombinant human N-ethylmaleimide-sensitive fusion protein (NSF) and α-soluble N-ethylmaleimide-sensitive fusion protein attachment protein (α-SNAP)^12–14^. Other PEN proteins in plants have been identified, including PEN2 (a β-glucosidase) and PEN3 (an ATP-binding cassette [ABC] transporter), which function together as a more broadly associated unit called the regulon^6,15–17^. Therefore, the regulon consists of numerous proteins, including 20 S particle components that act together during the defense response. Experiments have focused on examining shoot pathogens, but studies examining root pathogens are largely lacking^6,15,16,18,19^.

Among the many important root pathogens are plant parasitic nematodes, a devastating group among a much larger cohort of organisms that have significant global effects^20^. Recent experiments have examined the function of regulon proteins in plant roots in relation to root pathogens. These experiments employed the interaction between *Glycine max* (soybean) and the plant parasitic nematode *Heterodera glycines* (the soybean cyst nematode [SCN]) as a model. *H. glycines* is an obligate parasite, with *G*. *max* its primary host, that causes billions of dollars in economic losses every year^21,22^. Furthermore, *H. glycines* causes more economic losses than other *G. max* pathogens combined^22^. *G. max* may show obvious signs of *H. glycines* parasitism, such as chlorosis and stunting. However, complicating *H. glycines* detection, *G. max* may also show no adverse signs of parasitism except for an approximately 15% decrease in yield^23^. *H. glycines* also consists of a number of genetically distinctive variants, known as HG types, that relate in various ways to a race scheme^24–26^. Consequently, *G. max* cultivars that normally exhibit natural resistance to *H. glycines* can succumb to infection through processes dependent on the HG type with which it is infected. *H. glycines* has a life cycle of 30 or more days depending on ambient temperature^27^. Importantly, as part of its life cycle, *H. glycines* generates a hardened cyst formed from the carcass of the female containing 250–500 eggs. The eggs within these cysts can remain dormant in the soil for up to 9 years, complicating management practices. During successful parasitism of *G. max*, *H. glycines* eggs hatch as second-stage juveniles (J2s). The J2s migrate toward and then burrow into the root, subsequently slicing through epidermal, cortex and endodermal root cells with a rigid, tubular mouth apparatus known as a stylet. Upon reaching the root stele, the J2s use the stylet to deliver effectors into a *G. max* pericycle or neighboring cell. Over a period of days, the cell walls of the *H. glycines*-parasitized root cells dissolve through enzymatically driven processes mediated by the nematode. The outcome is the production of a multinucleate syncytium, the product of the incorporation of 200–250 neighboring root cells into a common cytoplasm^28,29^. The syncytium is also the site of the localized defense response^30^, which involves components of ETI and PTI^31–33^. However, for the purposes here, the term defense is used to refer to this localized defense response^15^.

A *G. max* ortholog of the 20 S particle vesicle transport apparatus component α-SNAP-5 (Glyma.18G022500) maps to the major *H. glycines* resistance locus (*rhg1*). The defense function of α-SNAP-5 has been shown through functional transgenic experiments^31^. Here, functional, transgenic experimental approaches included experimental increases in target gene expression through overexpression of the target gene in an *H. glycines*-susceptible *G. max* cultivar (*G. max*_[Williams 82/PI 518671]_), suppressing parasitism, and experimental decreases in target gene expression through RNA interference (RNAi) in an *H. glycines*-resistant *G. max* cultivar (*G. max*_[Peking/PI 548402]_), facilitating parasitism. The combination of experimentally suppressing *H. glycines* parasitism in *G. max*_[Williams 82/PI 518671]_ and experimentally facilitating *H. glycines* parasitism in *G. max*_[Peking/PI 548402]_ has demonstrated the roles of targeted genes, such as α-SNAP-5, in defense^31–34^. Complimentary studies have shown that an *H. glycines* effector directly binds α-SNAP-5^35^. Presumably, this nematode effector impairs or hijacks α-SNAP-5, interfering with its normal role during the *G. max* defense response. In this manner, the *H. glycines* effector generates effector-triggered susceptibility (ETS)^1^. These observations are similar to those in original studies showing that microbial effectors impaired SNARE protein function in animal systems using *R. norvegicus* and *Aplysia californica* (the sea slug) as models^36^. Those studies examining microbial pathogenesis showed that the microbial neurotoxin effectors botulinum from *Clostridium botulinum* and tetanus from *C. tetani* target SNARE components^36^. The interaction between botulinum and tetanus effectors inhibits secretion, resulting in paralysis^36^. These studies suggest that while *G. max* has a functional membrane fusion apparatus that it employs to impair *H. glycines* pathogenesis, nematode effectors target and perturb its function, facilitating parasitism^35^. These *H. glycines* effectors bind the *G. max* vesicle transport protein, presumably altering its function for the benefit of the parasite in ETS^35^.

The vesicle and target membrane fusion process consists of three steps: vesicle tethering to a target membrane, docking, and subsequent fusion of the vesicle and target membrane. The successful completion of this three-step membrane fusion process leads to the delivery of PRRs and release of the vesicular cargo. While components of the 20 S vesicle docking particle have been shown to play roles in defense against a number of pathogens, including *H. glycines*, in *G. max*, much less regarding the upstream process of tethering is known. In *S. cerevisiae*, a vesicle-bound protein known as Sec4p initiates tethering, and in *G. max,* a Sec4 homolog functions in defense against *H. glycines*^37^. *S. cerevisiae* Sec4p is a Rab GTPase that regulates the assembly of a structure called the exocyst^38,39^. This function of Sec4p in mediating tethering occurs through its interaction with the exocyst component Sec15p^38,39^. These results indicate that the *G. max* exocyst also performs a defense function against *H. glycines* parasitism.

The exocyst is an octamer composed of Sec3p (EXOC1), Sec5p (EXOC2), Sec6p (EXOC3), Sec8p (EXOC4), Sec10p (EXOC5), Sec15p (EXOC6), Exo70p (EXOC7) and Exo84p (EXOC8)^38,40–44^. The exocyst complex acts as a signal receiver for various signaling pathways^44,45^. Through this role, the exocyst helps tether vesicles at the receptor membrane and mediate fusion by inducing SNARE assembly^44,45^. Thus, tethering occurs upstream of the roles of the SNARE-containing 20 S particle in docking and fusion. The exocyst functions to promote a number of cellular processes. In general, these processes include exocytosis, cell polarity, growth, division, cell migration, ciliogenesis, autophagy, pollen compatibility and plant defense^8,18,44–47^. Relevant to plant defense is exocytosis, an evolutionarily conserved biological process that ultimately facilitates the fusion of secretory vesicles with a targeted membrane. Consequently, exocysts allow cells to deliver PRRs and cargo that may function in defense^8,18,44–47^.

Each exocyst component plays an important role in secretion. Mutants of *S. cerevisiae* Sec3 (*sec3*), the primary exocyst subunit that connects vesicles with the target membrane, exhibit secretory vesicle accumulation in the cytoplasm^48,49^ because vesicles are unable to tether with the target membrane^48,49^. *N. benthamiana* Sec5 (EXOC2) is important for secretion of the pathogenesis-related 1 (PR-1) protein and callose deposition in the process of defense against *Phytophthora infestans*^18^. *N. benthamiana* Sec5 is targeted by the *P. infestans* effector AVR1 RXLR, which impairs PR-1 secretion and callose deposition^18^. Notably, *G. max* PR-1 (Glyma.15G062400) functions in defense against *H. glycines*^50^. *G. max* PR-1 is also under regulation by mitogen-activated protein kinases (MAPKs)^50^. The work of Austin et al*.*^51^ led to the identification of a number of *G. max* callose synthases (CSs) expressed within the syncytium during the defense process with functions in defense. The results demonstrated that a secreted *G. max* protein (PR-1) and an enzyme (CS) that generates a secreted defense molecule (callose) function during the defense response against *H. glycines*^51^. Therefore, *G. max* PR-1 and CS act in a manner that is very similar to their function in *N. benthamiana* during PTI^11^. In another recent work, some pathogen effectors were shown to impair the function of the exocyst structure through ubiquitination of an exocyst protein component (Exo70B1) in a manner resembling ETS^52^. Due to the importance of each exocyst component, experiments have shown that the removal of just one protein impairs the ability of the other components to function properly^52–54^, resulting in the impairment of biological processes^52–54^.

Exocyst proteins are coiled-coil proteins that share some structural homology with helical bundles^41,55^. Helical bundles facilitate exocyst component interactions, which are essential for complex formation^41,55^. The structure of the exocyst complex is rod-shaped, with N- and C-termini located at opposite poles of the structure. This structure aids in the tethering of vesicles to the plasma membrane and delivery of vesicle cargo to the apoplast^38,40,44,46,56–58^. The exocyst functions by connecting vesicles through the EXOC5 and EXOC6 proteins to the plasma membrane through EXOC1 and EXOC7^38,44,49,59,60^. On the target (plasma) membrane is phosphatidylinositol 4,5-biphosphate (PI(4,5)P2), to which EXOC1 and EXOC7 bind^61–63^. In *S. cerevisiae*, the movement of vesicles is regulated by vesicle membrane-bound Sec4p, which directs the vesicle to the plasma membrane at a targeted site^64–67^. Through its interaction with EXOC6, Sec4p functions by regulating assembly of the exocyst^38,39^. These results support observations showing that *G. max* Sec4 functions in facilitating the defense response to *H. glycines*^37^.

The experiments presented here have identified the components of the *G. max* exocyst. At least one exocyst component of each gene family is expressed within the syncytium during the defense response of *G. max* against *H. glycines* parasitism. In some cases, these exocyst genes are under regulation by MAPKs. Experimental overexpression of exocyst genes in the *H. glycines*-susceptible cultivar *G. max*_[Williams 82/PI 518671]_ suppresses parasitism. In contrast, experimental decreases in the expression of exocyst components through RNAi in the *H. glycines*-resistant cultivar *G. max*_[Peking/Pi 548402]_ facilitate parasitism. The combination of suppressing *H. glycines* parasitism in a normally susceptible *G. max* cultivar and facilitating *H. glycines* parasitism in a normally resistant *G. max* cultivar successfully demonstrated the functions of the target genes in defense. These results demonstrate that the *G. max* exocyst plays an important role in defense against *H. glycines* parasitism. Furthermore, these results show the importance of the plant secretion process to defense in general.

## Results

### Exocyst genes were expressed within *H. glycines*-parasitized root cells during defense

The observation that *G. max* Sec4 functions in defense against *H. glycines* parasitism implies a similar role for the exocyst^37^. The most recently released *G. max* genome annotation (Wm82.a2.v1) was examined through BLAST searches using *A. thaliana* exocyst component protein sequences as a query. The analysis resulted in the identification of 5 EXOC1 genes, 2 EXOC2 genes, 5 EXOC3 genes, 2 EXOC4 genes, 2 EXOC5 genes, 6 EXOC6 genes, 31 EXOC7 genes and 8 EXOC8 genes (Supplementary Table S1). These gene accessions served as the basis for subsequent analyses.

**Figure 1:**
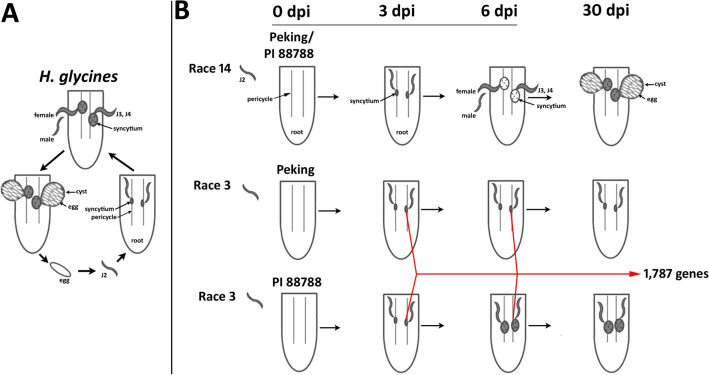
*H. glycines* life cycle and gene identification. (**a**) Egg; juvenile stages, second (J2), third (J3), and fourth (J4). Male, female; the parasitized pericycle cells develop into a syncytium, a nurse cell structure composed of the merged cytoplasm from 200 to 250 cells that serves as the site of the localized defense response; cyst, female carcass structure containing the eggs. (**b**) From the experiments published by Matsye et al*.*^31^, the *G. max*_[Peking/PI 548402]_ and *G. max*_[PI 88788]_ cultivars are susceptible to infection with *H. glycines*_[race 14/HG-type 1.3.6.7/TN8]_, while infection with *H. glycines*_[NL1-Rhg/HG-type 7/race 3]_ leads to a resistant reaction. The defense response in *G. max*_[Peking/PI 548402]_ arrests *H. glycines*_[NL1-Rhg/HG-type 7/race 3]_ development earlier (the J2 stage), resulting in a smaller syncytium (gray). The defense response in *G. max*_[PI 88788]_ arrests *H. glycines*_[NL1-Rhg/HG-type 7/race 3]_ development later (the J3–J4 stages), resulting in a larger syncytium that eventually becomes nonfunctional and acts as the site of the defense response (gray). The *H. glycines* life cycle is completed at approximately 30 dpi, a time point that was not a part of the gene expression studies. Matsye et al*.*^31^ identified 1,787 genes expressed specifically within the syncytium during the defense response in *G. max*_[Peking/PI 548402]_ and *G. max*_[PI 88788]_ infected with *H. glycines*_[NL1-Rhg/HG-type 7/race 3]_; these genes were identified in syncytia isolated from roots infected at 3 and 6 days postinfection (dpi) but not pericycle control cells. These genes are the focus of the study presented here.

Here, the *H. glycines* life cycle guided the design of gene expression experiments (Fig. 1)^31^ employing LM to isolate RNA from targeted cells. The targeted cells are involved in successful parasitism by *H. glycines* during a susceptible reaction and the defense response by *G. max* during a resistant reaction. The collected cells included pericycle cells and surrounding cells and were collected at 0 dpi. Furthermore, syncytia were collected at an early stage of parasitism (3 dpi). Syncytia formed in susceptible or resistant reactions at 3 dpi showed a similar cytological appearance. Their features included hypertrophy, the enlargement of nuclei, the development of dense cytoplasm and an increase in the endoplasmic reticulum (ER) and ribosome content. As a consequence of these similarities, 6 dpi was selected as a time point. The 6-dpi time point assisted in differentiating between a susceptible and resistant reaction. By 6 dpi, the syncytia formed during a susceptible reaction were characterized by the hypertrophy of nuclei and nucleoli, proliferation of cytoplasmic organelles, a reduction in vacuoles, the dissolution of vacuoles and cell expansion due to the incorporation of adjacent cells. In contrast, the cytoplasmic characteristics of the resistant reaction were genotype-specific. For example, by 6 dpi, *G. max*_[Peking/PI 548402]_ showed cell wall apposition, structures consisting of cytoplasmic components that aggregated through actin polarization and the vesicle-mediated delivery of cargo. Furthermore, the *G. max*_[Peking/PI 548402]_ defense response at 6 dpi included the production of a necrotic layer of cells surrounding the syncytium and the accumulation of ER, which led *H. glycines* development to be blocked at the parasitic J2 stage. In contrast, the *G. max*_[PI 88788]_ defense response was not characterized by cell wall apposition or a necrotic layer of cells surrounding the syncytium during the resistance reaction, but the ER had accumulated, leading to the blockade of *H. glycines* development at the J3–J4 stage (Fig. 1)^31^. The cDNA probe made from mRNA^31^ was tagged with a proprietary Affymetrix label and used for gene expression studies, leading to the identification of a pool of 1,787 candidate defense genes (Fig. 1)^31^.

**Figure 2:**
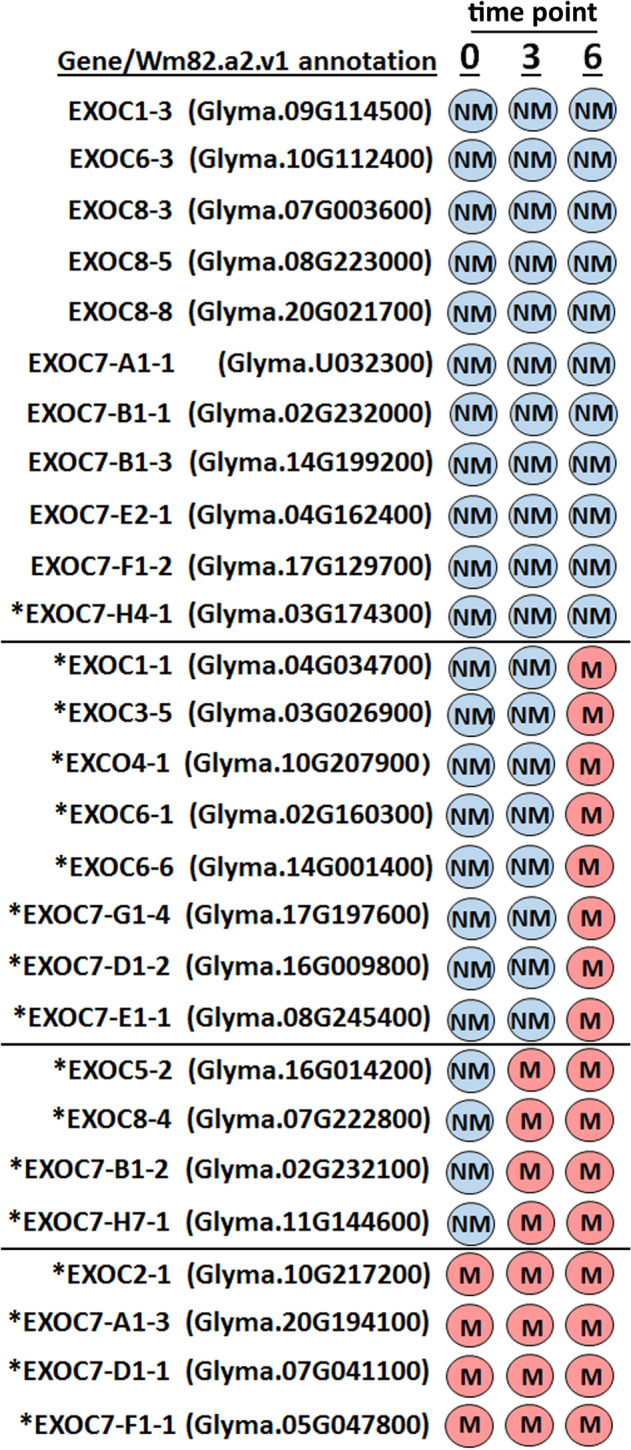
Expression of the exocyst genes within the syncytium. The exocyst genes are expressed in the syncytium during the defense response in *G. max*_[Peking/PI 548402]_ and *G. max*_[Peking/PI 88788_], as shown by detection call methodology (DCM)^31^. Please refer to the Materials and Methods (“Exocyst genes were expressed within *H. glycines*-parasitized root cells during defense” section) for details of the analysis. Blue, expression was measured (NM) by DCM (*p* ≥ 0.05). Red, gene expression was measured (M) by DCM (*p* < 0.05).

This analysis was focused on examining the relationship between *G. max* and its defense response against *H. glycines* parasitism in relation to the exocyst (Fig. 1). From these data, exocyst genes expressed within the syncytium were identified (Fig. 2). Under our analytical parameters, the exocyst genes exhibited four profiles of expression in relation to their defense response to *H. glycines*. However, other gene expression profiles not observed here are possible. First, 11 exocyst genes were not expressed at any time point: EXOC1-3, EXOC6-3, EXOC8-3, EXOC8-5, EXOC8-8, EXOC7-A1-1, EXOC7-B1-1, EXOC7-B1-3, EXOC7-E2-1, EXOC7-F1-2 and EXOC7-H4-1 (Fig. 2). Second, 8 exocyst genes lacked measurable gene expression at 0 dpi (control) but were expressed at the 6-dpi time point: EXOC1-1, EXOC3-5, EXOC4-1, EXOC6-1, EXOC6-6, EXOC7-D1-2, EXOC7-E1-1 and EXOC7-G1-4 (Fig. 2). The third group was composed of 4 exocyst genes expressed at only 3 and 6 dpi: EXOC5-2, EXOC8-4, EXOC7-B1-2 and EXOC7-H7-1 (Fig. 2). The fourth group of 4 exocyst genes was expressed at all three time points (0, 3 and 6 dpi): EXOC2-1, EXOC7-A1-3, EXOC7-D1-1 and EXOC7-F1-1 (Fig. 2). Consequently, the results revealed that 16 different exocyst genes were expressed in samples from at least one of the time points chosen for the analysis. Furthermore, the analyses identified a component of each exocyst gene family expressed within cells parasitized by *H. glycines* as the root cell underwent a defense response. Last, among these 16 exocyst genes, four were expressed at the 0-dpi time point, 8 were expressed by the 3-dpi time point, and 16 were expressed by the 6-dpi time point. These results demonstrate an increase in the number of exocyst genes expressed during the course of the defense process. Notably, expression of a number of exocyst genes in root cells could not be evaluated (n/a) due to the nature of the original root cell gene expression analyses (Supplementary Table S2)^31^. While not studied in the functional analyses presented in “Functional analysis of syncytium-expressing exocyst genes demonstrates a defense role” section, the expression of those exocyst genes was examined in transcriptomic analyses of defense MAPKs, the results of which are presented in “The expression of certain exocyst genes was induced by specific defense MAPKs” section^50^. An additional examination determined whether the exocyst genes are regulated by signaling processes in the *G. max* defense against *H. glycines* and may also be of interest here^50^.

### The expression of certain exocyst genes was induced by specific defense MAPKs

Recent experiments identified a subset of 9 (of the 32) *G. max* MAPKs whose experimentally induced expression in the normally *H. glycines*-susceptible cultivar *G. max*_[Williams 82/PI 518671]_, resulted in an engineered defense response to *H. glycines*^50^. In contrast, experimental suppression of the expression of the same 9 defense MAPKs by RNAi in the normally *H. glycines*-resistant *G. max*_[Peking/PI 548402]_ cultivar impaired the defense response^50^. RNA from these defense MAPK-OE and RNAi transgenic lines was subjected to RNA-seq analyses^68^, which led to the identification of thousands of transcripts whose relative abundances either increased or decreased^68^. Consequently, the *G. max* exocyst gene family as a whole was examined here via transcriptomic analyses of those MAPK-OE and MAPK-RNAi lines. Analyses of those RNA-seq data was conducted to determine if the syncytium-expressed exocyst genes was also expressed within individual MAPK-OE or MAPK-RNAi lines^50^. Further, the analysis also determined whether the exocyst genes were expressed across many lines overexpressing the defense MAPKs^50^. The results demonstrated that the differential expression of exocyst genes was primarily found in specific transgenic MAPK lines (Supplementary Table S2). For many exocyst genes, differential expression was not observed at all (Supplementary Table S2). However, even if an exocyst gene was not differentially expressed (NDE), this does not mean that the gene is not expressed at all (lacking identified sequences in the RNA-seq studies).

**Figure 3:**
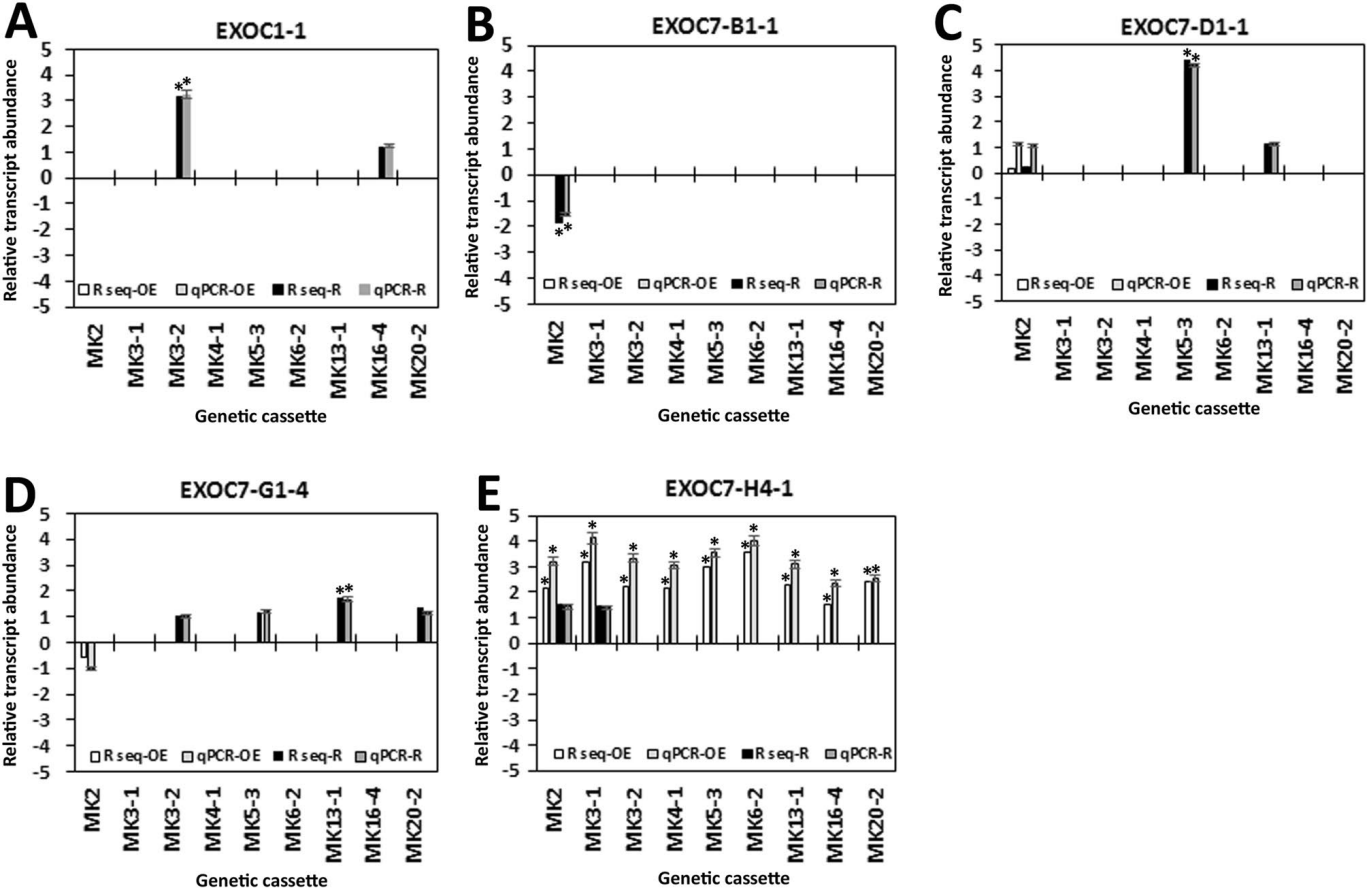
qRT-PCR confirmation of relative changes in transcript abundance of the exocyst subunit in the MAPK-OE and MAPK-RNAi transgenic roots first shown by RNA-seq analysis. In cases in which a numerical value of differential expression was obtained in the RNA-seq analyses (not necessarily ± 1.5-fold) and the *p *value was < 0.05 (*p* < 0.05), qRT-PCR was employed to confirm the relative change in transcript abundance of the exocyst gene of interest. These results were compared to the RPS21 control employing the 2^−ΔΔ*CT*^ method^31,69,70^. For a change in relative transcript abundance to be considered statistically significant, a minimum cutoff of ± 1.5-fold was set, and *p* < 0.05. The *p* values for the replicate qRT-PCR analyses were calculated through Student’s *t* test^70^. Error bars represent the standard deviation. Please refer to the Materials and Methods (“Functional analysis of a MAPK-induced exocyst genes that were not expressed in the syncytium” section) for details of the analysis.

Among the exocyst genes inducing MAPK overexpression, EXOC7-H4-1 and EXOC7-H7-1 exhibited higher relative transcript levels in all 9 defense MAPK-OE lines. However, when their expression within the syncytium was examined, EXOC7-H4-1 lacked expression in the 0-dpi control samples as well as the 3-dpi and 6-dpi samples from syncytia during the defense response (Fig. 2). While EXOC7-E2-1 was also not expressed in the 0-dpi control samples or 3-dpi or 6-dpi syncytium samples, was expressed at almost the same level as EXOC7-H4-1in the defense MAPK-OE lines. However, EXOC7-E2-1 gene expression was observed in just 8 of the 9 defense MAPK-OE lines. EXOC7-E2-1 was not further examined via qRT-PCR or functional studies since it was not expressed in the syncytium or in all 9 of the defense MAPK-OE lines. In contrast, EXOC7-H7-1 was expressed in the 3-dpi and 6-dpi samples from syncytia during the defense response but not in the 0-dpi samples (Fig. 2). Expression of the exocyst genes that showed expression in the parasitized root cells as well as some of the transgenic MAPK-OE or MAPK-RNAi lines was confirmed in the transgenic MAPK-OE and MAPK-RNAi lines. Their RNA-seq expression data were confirmed by qRT-PCR using the RPS21 gene as a control. These 4 exocyst genes were EXOC1-1, EXOC7-B1-1, EXOC7-D1-1 and EXOC7-G1-4 (Fig. 3). During the course of the analysis, a single exocyst gene (EXOC7-H4-1) was found not to be expressed within the syncytium. However, the increased expression of EXOC7-H4-1 in all 9 of the MAPK-OE lines was confirmed by qRT-PCR using the RPS21 gene as a control (Fig. 3). The observation that EXOC7-H4-1 expression was not measured within the syncytium but was increased in all 9 of the defense MAPK-OE lines (MAPK-all-OE) is notable. This result indicates that processes involving the *G. max* secretion apparatus outside the vicinity of the syncytium are important to defense. This hypothesis was examined later in the analysis.

A number of exocyst genes showed increased expression in the MAPK-OE lines. However, their expression in the parasitized root cells could not be measured by the DCM analysis presented here because probe sets corresponding to those genes were lacking on the Affymetrix microarray. These genes were EXOC1-2, EXOC3-4, EXOC8-7, EXOC7-C2-2, EXOC7-E2-2, EXOC7-G1-1, EXOC7-G1-3, EXOC7-H4-2, EXOC7-H4-3, EXOC7-H7-2, EXOC7-H7-4 and EXOC7-H7-5 (Supplementary Table S2). The RNA-seq gene expression data revealed that the differential expression of some of these exocyst genes was observed in many of the different transgenic MAPK-OE lines. The exocyst genes expressed in two or more different transgenic MAPK-OE lines were EXOC1-2 (4 lines), EXOC3-4 (4 lines), EXOC7-C2-2 (5 lines), EXOC7-E2-1 (8 lines), EXOC7-E2-2 (6 lines), EXOC7-H4-2 (2 lines), EXOC7-H4-3 (13 lines), EXOC7-H7-2 (8 lines), and EXOC7-H7-5 (2 lines). In contrast, some of the RNA-seq expression data revealed that the differential expression of some of these exocyst genes was limited. In these cases, the exocyst gene was expressed in one transgenic MAPK-OE line (Supplementary Table S2). The exocyst genes expressed in one transgenic line were EXOC1-2, EXOC8-7, EXOC7-G1-1 and EXOC7-H7-4 (Supplementary Table S2). Examination of exocyst genes whose expression was not measured in the parasitized root cells by DCM (n/a) is beyond the scope of this study (Supplementary Table S2). Consequently, these exocyst genes were not further examined (Supplementary Table S2). Furthermore, examination of the relationships between other types of signaling processes that may occur and the exocyst is beyond the scope of this analysis.

### Transgenic plants showed the expected effect on EXOC gene expression

**Figure 4:**
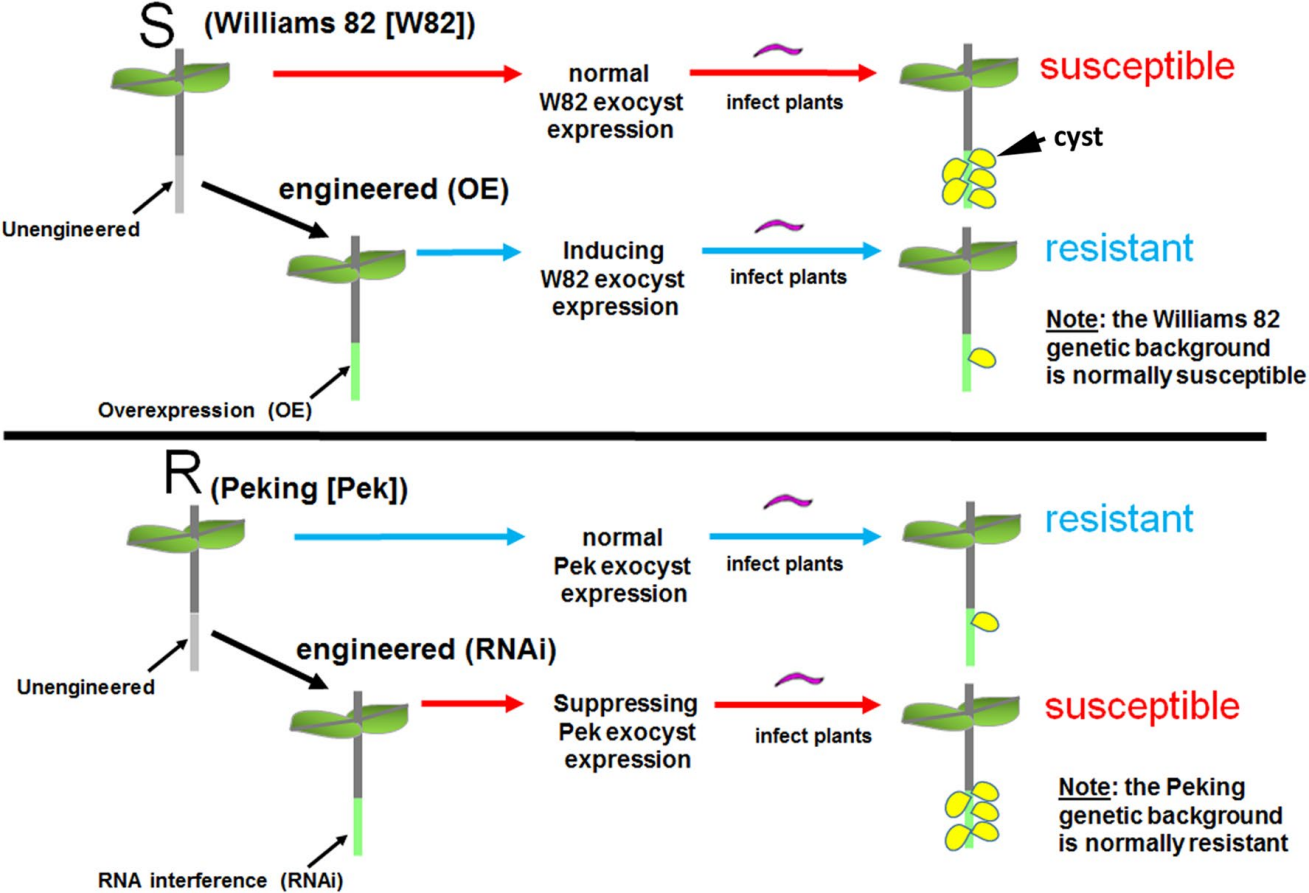
Analysis pipeline. (**a**) *H. glycines*-susceptible (S) *G. max*_[Williams 82/PI 518671]_ (W82) (gray) was engineered to overexpress an exocyst component, as visualized by the reporter eGFP (green). Transgenic pRAP15-expressing control roots and roots overexpressing an exocyst gene were infected by *H. glycines* (magenta). After 30 days, the cysts (yellow) were extracted and enumerated with respect to the whole root (wr) system and cysts per gram (pg) of root system, resulting in a calculated female index (FI). (**b**) *H. glycines*-resistant (R) *G. max*_[Peking/PI 548402]_ (Pek) roots were engineered to undergo RNAi of an exocyst component, as visualized by the reporter eGFP. Transgenic pRAP17-expressing control roots and roots undergoing RNAi targeting of the mRNA of an expressed exocyst gene were infected by *H. glycines* (magenta). After 30 days, the cysts were extracted and enumerated with respect to the wr system and cysts pg of root system, resulting in a calculated FI. Please refer to the Materials section (“Functional analysis of a MAPK-induced exocyst genes that were not expressed in the syncytium” section) for details.

**Figure 5:**
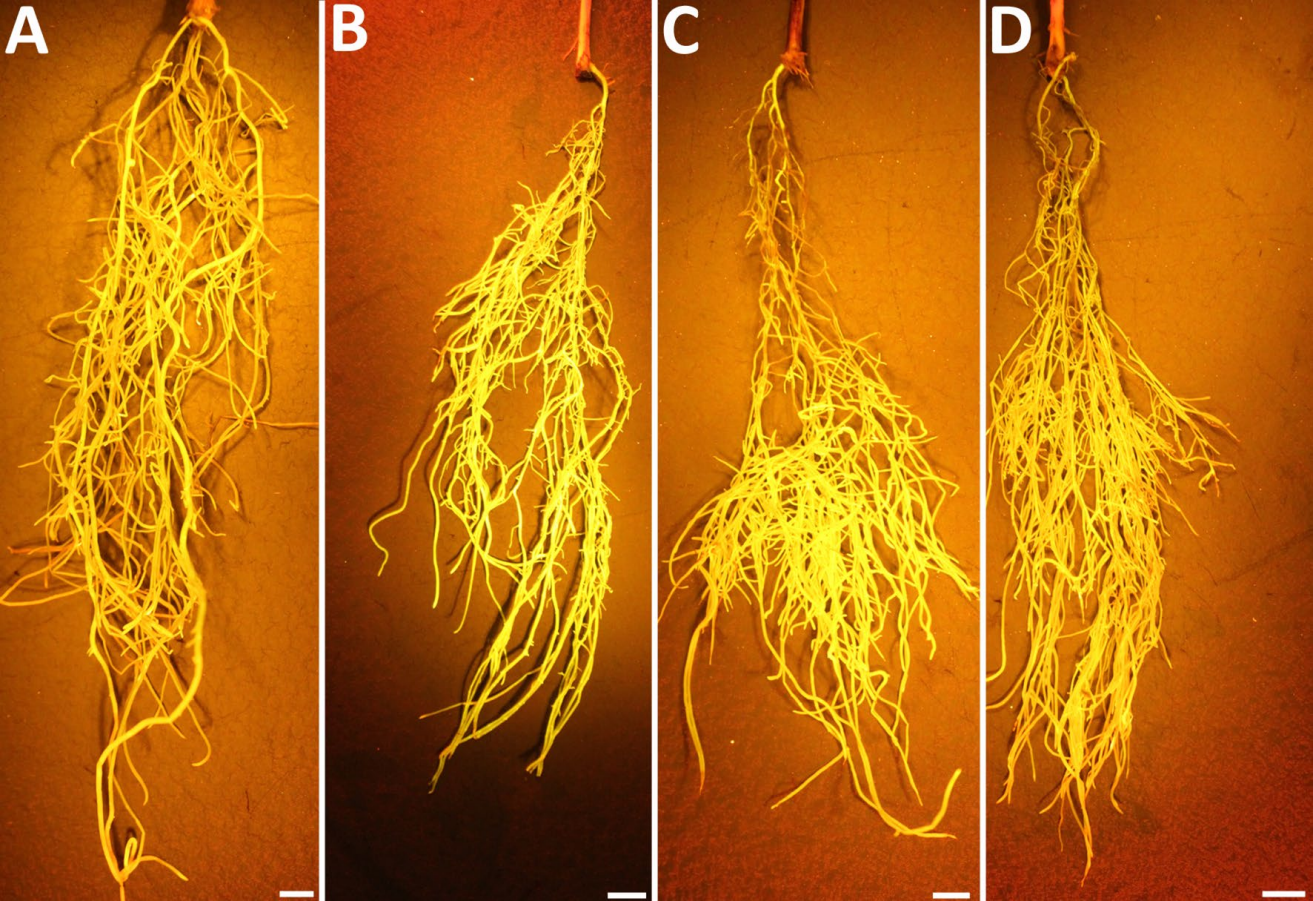
Transgenic roots. (**a**) pRAP15-OE control. (**b**) A representative OE-expressing root (EXOC1-1). (**c**) pRAP17-RNAi control. (**d**) RNAi-expressing root (EXOC1-1). The roots were illuminated with the Dark Reader Spot Lamp as described in the Materials and Methods (“Functional analysis of syncytium-expressing exocyst genes demonstrates a defense role” section). Bar = 1 cm.

**Figure 6:**
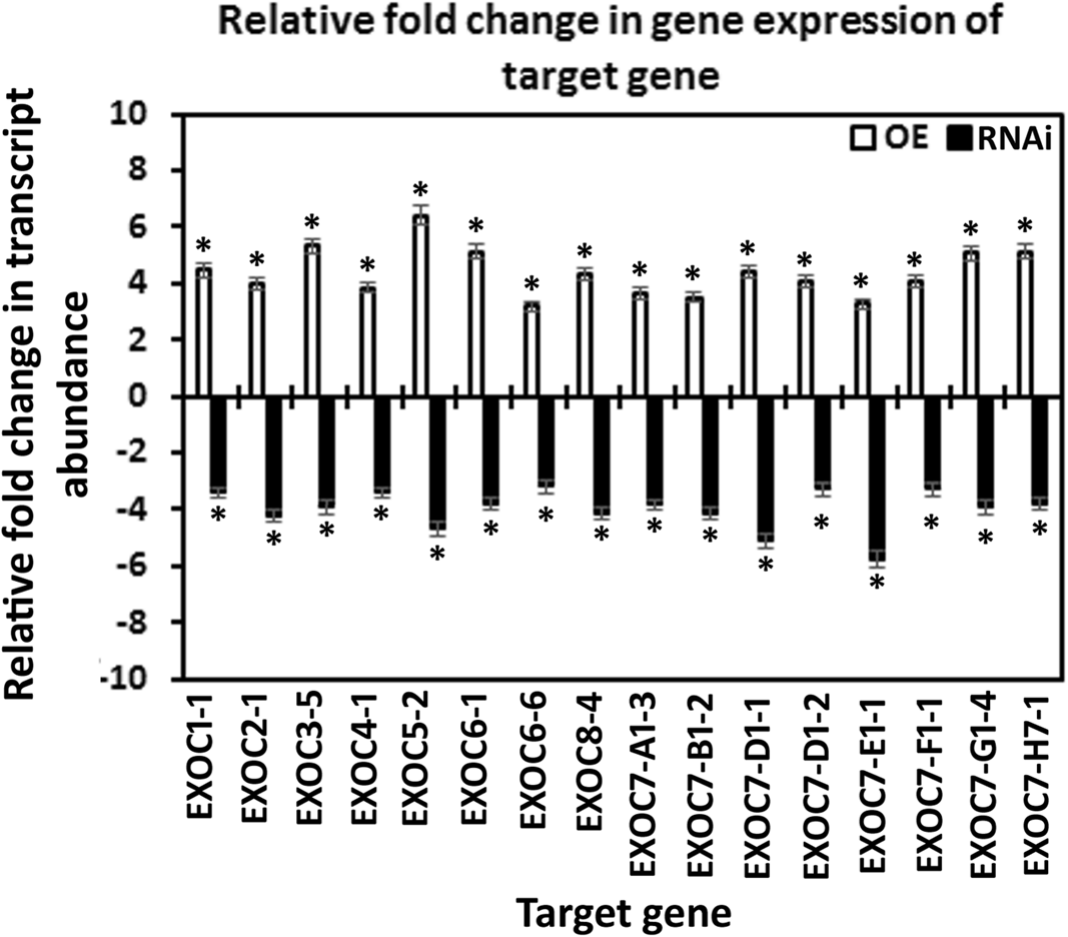
qRT-PCR confirmation of the OE of syncytium-expressed exocyst genes and RNAi of these genes through gene cassettes in transgenic roots. The results were compared to those in the RPS21 control employing the 2^−ΔΔ*CT*^ method^31,69,70^. A minimum cutoff of ± 1.5-fold was set, and *p* < 0.05. *Statistically significant. The *p* values for the replicate qRT-PCR analyses were calculated through Student’s *t* test^70^. Error bars represent the standard deviation. Please refer to the Materials and Methods (“Functional analysis of a MAPK-induced exocyst genes that were not expressed in the syncytium” section) for details of the analysis.

**Figure 7:**
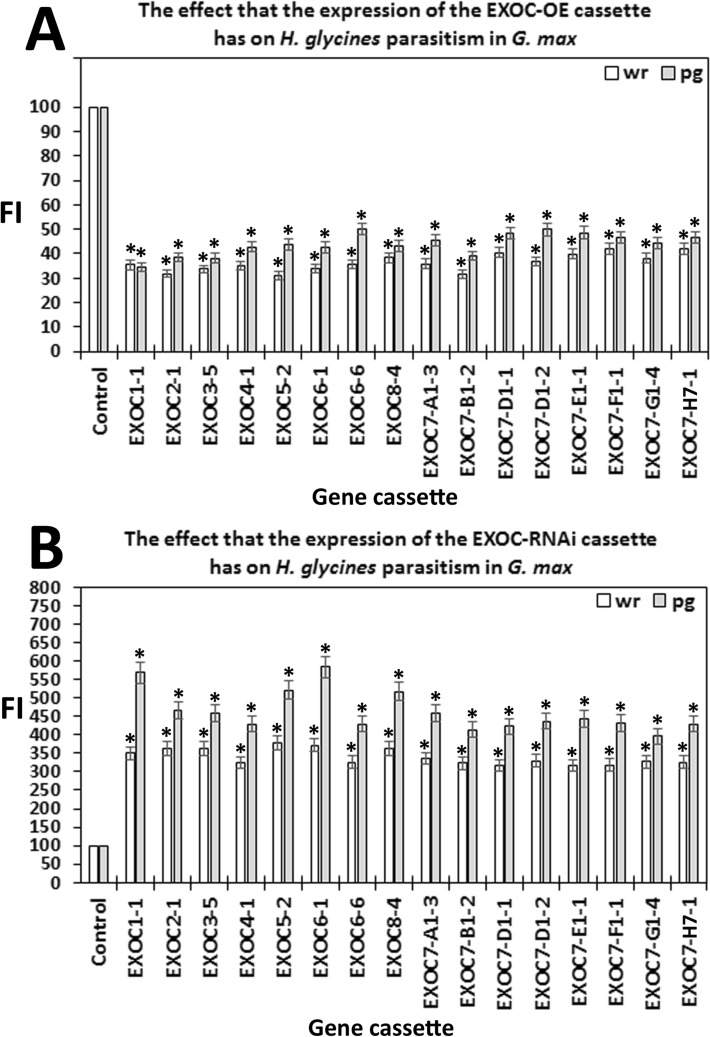
The effect of each transgene on root mass is presented as a percent compared to the control. The root weight for OE analysis of the control (*G. max*_[Williams 82/PI 518671]_) was 4.24 ± 0.5 g. The root weight for RNAi analysis of the control (*G. max*_[Peking/PI 548402]_) was 2.87 ± 0.53 g. The values for the EXOC genes in determined by the respective OE and RNAi analyses were not statistically significant (*p* ≥ 0.05). The *p* values for the replicate root masses analyses were calculated through Student’s *t* test^70^.

Affymetrix DCM microarray analysis showed that exocyst genes were expressed within parasitized root cells (syncytia) during the defense response. These 16 exocyst genes were EXOC1-1, EXOC3-5, EXCO4-1, EXOC6-1, EXOC6-6, EXOC7-G1-4, EXOC7-D1-2, EXOC7-E1-1, EXOC5-2, EXOC8-4, EXOC7-B1-2, EXOC7-H7-1, EXOC2-1, EXOC7-A1-3, EXOC7-D1-1 and EXOC7-F1-1. The 16 exocyst genes were cloned and functionally tested through transgenic analyses. Functional transgenic tests of the exocyst genes were conducted to determine if they play a role in defense. The genes were overexpressed in the *H. glycines*-susceptible *G. max*_[Williams 82/PI 518671]_ cultivar, after which whether the *H. glycines*-susceptible cultivar became resistant to parasitism was determined (Fig. 4). In contrast, the same genes were engineered as RNAi cassettes used to decrease their expression in the *H. glycines*-resistant *G. max*_[Peking/PI 548402]_ cultivar. These functional transgenic tests were conducted to determine whether decreased exocyst gene expression would result in *H. glycines* susceptibility (Fig. 4). A combination of two outcomes had to be met for a gene to meet our criteria for a defense role^33^. First, *H. glycines* parasitism had to be decreased when the exocyst gene was overexpressed in *H. glycines*-susceptible *G. max*_[Williams 82/PI 518671]_. Second, *H. glycines* parasitism had to be increased when the gene was knocked down by RNAi in *H. glycines*-resistant *G. max*_[Peking/PI 548402]_. Transgenic plants in which genes were overexpressed or knocked down via RNAi were shown by expression of the eGFP reporter (Fig. 5). Furthermore, to confirm that the expression cassettes functioned as expected, qRT-PCR showed that the relative transcript abundance of the exocyst components was increased in the overexpression lines and decreased in the RNAi lines in comparison to that in the RPS21 gene-expressing control (Fig. 6). The effect of expression of the transgene cassette on root mass was then analyzed. The results demonstrated that expression of the cassettes did not have a statistically significant effect on root mass when the data were compared to those in the respective controls (*p* < 0.05) (Fig. 7). However, effects on only root mass were considered in this analysis.

### Functional analysis of syncytium-expressing exocyst genes demonstrates a defense role

The effect of altered exocyst gene expression on *H. glycines* parasitism was tested. The genetically engineered *G. max* roots were infected with *H. glycines*_[NL1-Rhg/HG-type 7/race 3]_ as described in the Materials and Methods section (“Assaying the effect the genetic engineering events on nematode parasitism” section). The data from the experimental replicates were compared to those from the corresponding controls in the overexpression (pRAP15-*ccd*B control) and RNAi (pRAP17-*ccd*B control) studies.

In the first set of analyses, engineering of the pRAP15-*ccd*B vector in *H. glycines*-susceptible *G. max*_[Williams 82/PI 518671]_ produced a robust level of infection. Quantification of the level of infection showed a cyst count of 203.09 ± 5.04 cysts per wr system and 49.03 ± 5.07 cysts pg of root system. All exocyst-OE transgenic lines were compared to this standard run in triplicate (please refer to Materials and Methods section “Assaying the effect the genetic engineering events on nematode parasitism” section for details of the analysis.). In contrast, engineering of the pRAP17-*ccd*B control vector in *H. glycines*-resistant *G. max*_[Peking/PI 458402]_ strongly suppressed parasitism. Quantification of the level of infection showed 9.94 ± 1.3 cysts per wr system and 2.52 ± 0.43 cysts pg of root system. All exocyst-RNAi transgenic lines were compared to this standard run in triplicate (please refer to Materials and Methods section “Assaying the effect the genetic engineering events on nematode parasitism” section for details of the analysis.).

**Figure 8:**
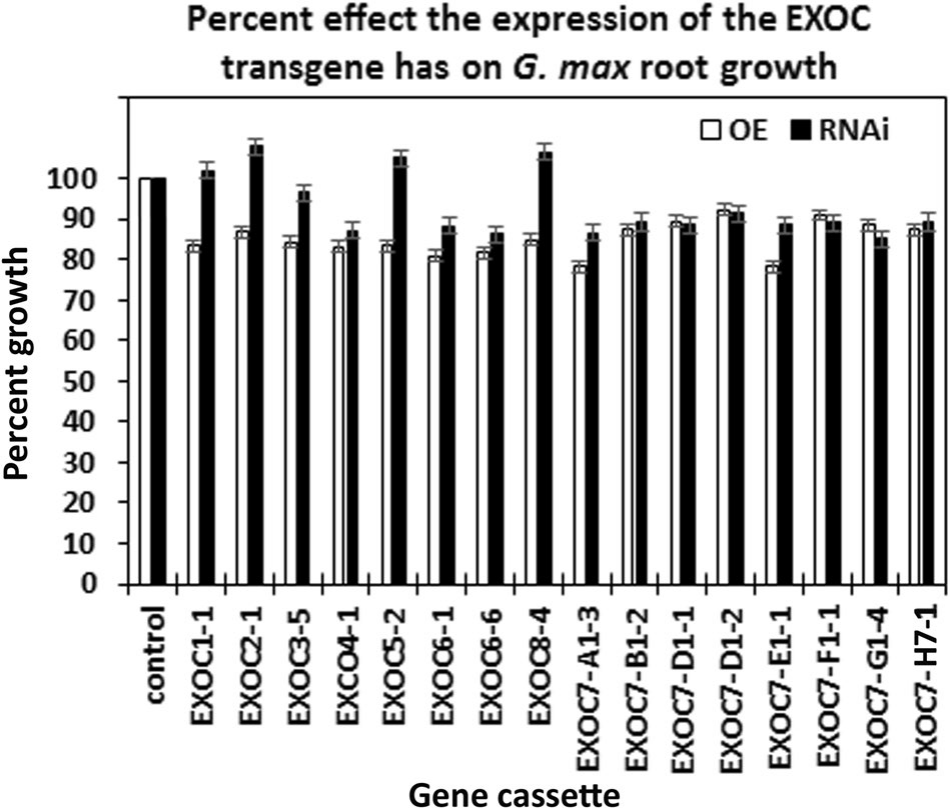
Functional analyses demonstrated that genetic engineering of the exocyst gene had the expected effect in the *G. max* root infected by *H. glycines*, as calculated by the FIs for the OE and RNAi lines compared to those of the controls with regard to the cysts per whole root (wr) system and per gram (pg) of root system. The cyst count for the OE analysis of the control (*G. max*_[Williams 82/PI 518671]_) was 203.09 ± 5.04 cysts per wr system and 49.03 ± 5.07 cysts pg of root system. The cyst count for RNAi analysis of the control (*G. max*_[Peking/PI 548402]_) was 9.94 ± 1.3 cysts per wr system and 2.52 ± 0.43 cysts pg of root system. The values for the EXOC genes in the respective OE and RNAi analyses were statistically significant, as indicated in the figure. *Statistically significant (*p* < 0.05), as calculated by the Mann–Whitney–Wilcoxon (MWW) rank-sum test^71^. The experimental error representing the standard deviation is presented. The results are the average of three independently run biological replicates; all *p *values < 0.001. Please refer to the Materials section (“Functional analysis of a MAPK-induced exocyst genes that were not expressed in the syncytium” section) for details of the analysis.

The second set of analyses focused on syncytium-expressing exocyst components. Calculation of the FI showed that *H. glycines* parasitism was significantly reduced by 58–68% in roots overexpressing each of the exocyst genes in cysts per wr system and by 50–64% in cysts pg of root system (both *p *values < 0.001) (Fig. 8). Consequently, the overexpression of exocyst components in the *H. glycines*-susceptible *G. max*_[Williams 82/PI 518671]_ cultivar decreased its susceptibility to *H. glycines* in comparison to that of the respective control. In contrast, RNAi of the candidate exocyst defense genes in *G. max*_[Peking/PI 548402]_ increased *H. glycines* parasitism by 3.16–3.77 times in the wr and 4.13–5.68 times pg of root system (both *p *values < 0.001) (Fig. 8). Consequently, RNAi of the exocyst components increased susceptibility of the *H. glycines*-resistant *G. max*_[Peking/PI 458402]_ cultivar to *H. glycines* in comparison to that of the respective control.

### Functional analysis of a MAPK-induced exocyst genes that were not expressed in the syncytium

**Figure 9:**
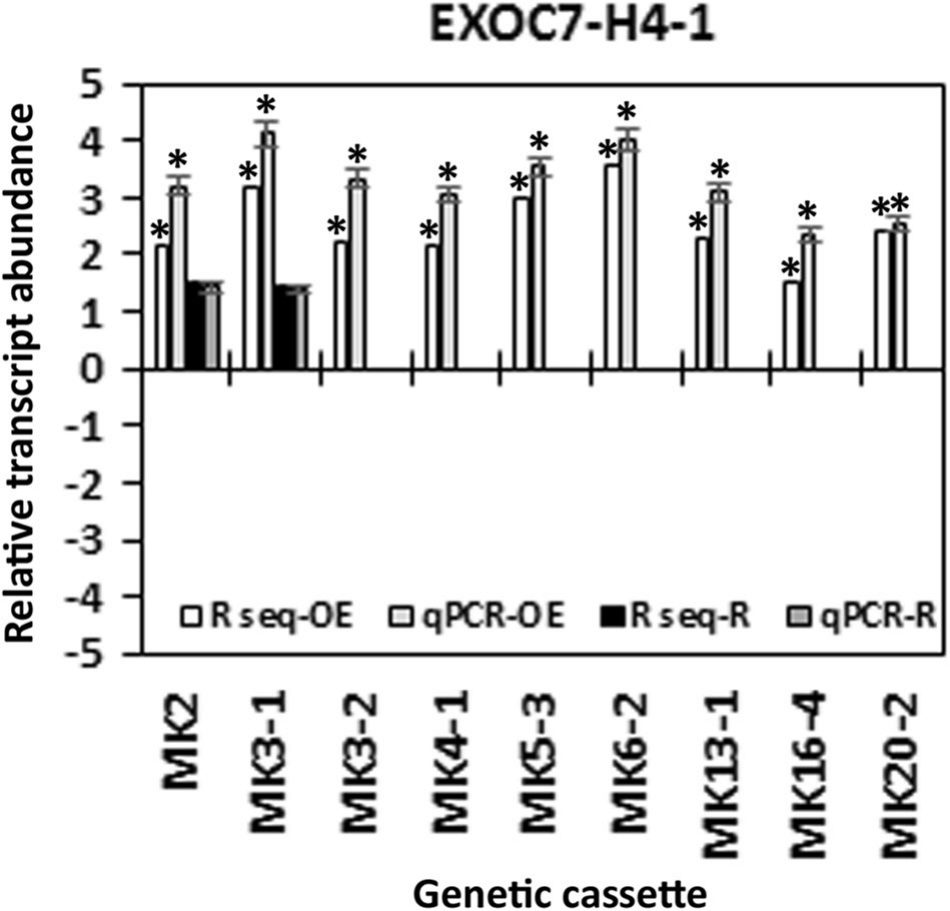
qRT-PCR confirmation of the relative change in EXOC7-H4-1 transcript abundance in MAPK-OE and MAPK-RNAi transgenic roots first analyzed by RNA-seq. In cases in which a numeric value indicating differential expression was obtained by the RNA-seq analyses and the *p* value was < 0.05 (*p* < 0.05), qRT-PCR is employed to confirm the relative change in transcript abundance. The results were compared to those in the RPS21 control employing the 2^−ΔΔ*CT*^ method^31,50,69^. For a change in relative transcript abundance to be considered statistically significant, a minimum cutoff of ± 1.5-fold was set, and *p* < 0.05. *Statistically significant. The *p* values for the replicate qRT-PCR analyses were calculated through Student’s *t* test^70^. Error bars represent the standard deviation. Please refer to the Materials and Methods (“Functional analysis of a MAPK-induced exocyst genes that were not expressed in the syncytium” section) for details of the analysis.

**Figure 10:**
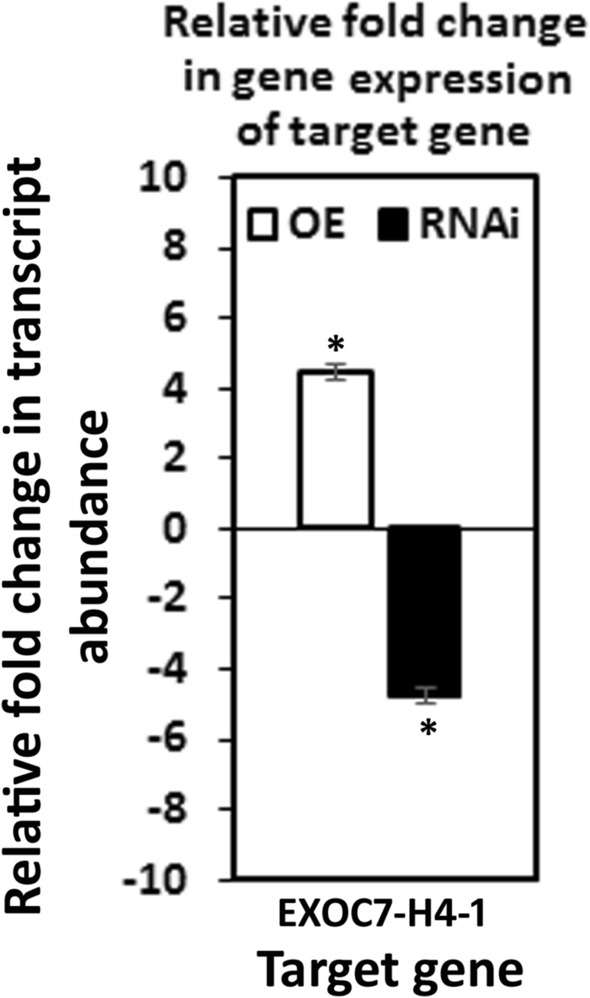
qRT-PCR confirmation of EXOC7-H4-1 exocyst gene OE and RNAi through gene cassette expression in transgenic roots; EXOC7-H4-1 expression was induced by MAPKs but not observed within the syncytium, as determined in the DCM analyses. The results were compared to those in the RPS21 control employing the 2^−ΔΔ*CT*^ method^31,50,69^. A minimum cutoff of ± 1.5-fold was set, and *p* < 0.05. *Statistically significant. The *p *values for the replicate qRT-PCR analyses were calculated through Student’s *t *test^70^. Error bars represent the standard deviation. Please refer to the Materials and Methods (“Functional analysis of a MAPK-induced exocyst genes that were not expressed in the syncytium” section) for details of the analysis.

**Figure 11:**
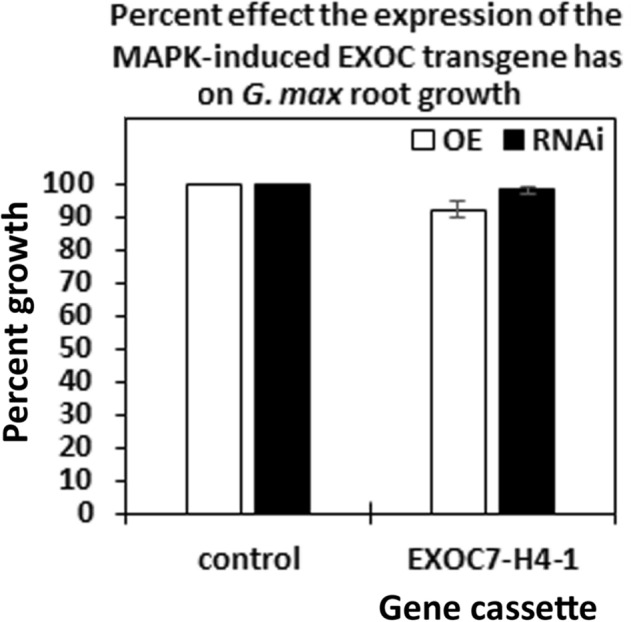
The effect of the EXOC7-H4-1 transgene on root mass is presented as a percent compared to the root mass of the control. The root weight for OE analysis of the control (*G. max*_[Williams 82/PI 518671]_) was 4.24 ± 0.5 g. The root weight for RNAi analysis of the control (*G. max*_[Peking/PI 548402]_) was 2.87 ± 0.53 g. The values for the EXOC7-H4-1 gene in the respective OE and RNAi analyses were not statistically significant (*p* ≥ 0.05). The *p* values for the replicate root mass analyses were calculated through Student’s *t *test^70^.

**Figure 12:**
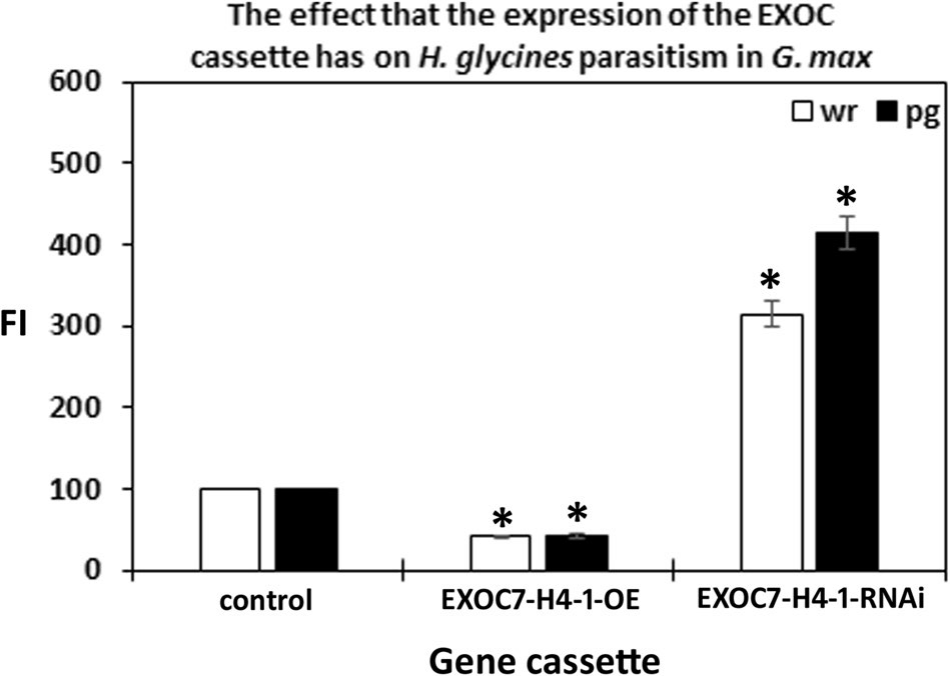
Functional analyses demonstrated that genetic engineering of the candidate defense exocyst gene EXOC7-H4-1 had the expected effect on *G. max* defense against *H. glycines*. The calculated FIs for the OE and RNAi lines compared to the controls are shown with regard to the cysts per whole root (wr) system and per gram (pg) of root. The cyst count for OE analysis of the control (*G. max*_[Williams 82/PI 518671]_) was 203.09 ± 5.04 cysts per wr system and 49.03 ± 5.07 cysts pg of root. The cyst count for the RNAi analysis of the control (*G. max*_[Peking/PI 548402]_) was 9.94 ± 1.3 cysts per wr system and 2.52 ± 0.43 cysts pg of root. The values for the EXOC genes in their respective OE and RNAi analyses were statistically significant, as indicated in the figure. *Statistically significant, *p* < 0.05, as calculated by the Mann–Whitney–Wilcoxon (MWW) rank-sum test^71^. The experimental error representing the standard deviation is presented. The results are the average of three independently run biological replicates; all *p *values < 0.001. Please refer to the Materials and Methods section (“Functional analysis of a MAPK-induced exocyst genes that were not expressed in the syncytium” section) for details of the analysis.

**Figure 13:**
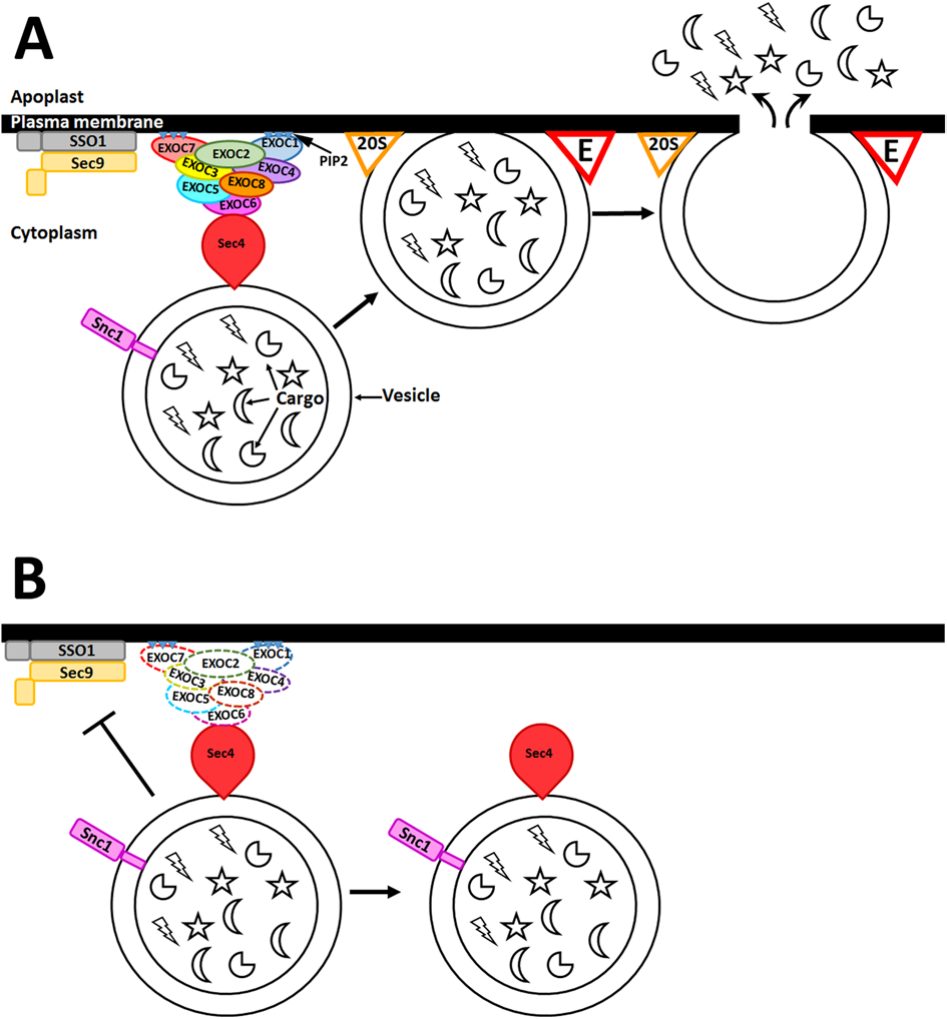
Model of the function of the exocyst complex during defense. (**a**) The exocyst is composed of 8 subunits: EXOC1, EXOC2, EXOC3, EXOC4, EXOC5 and EXOC6, EXOC7 and EXOC8. In *S. cerevisiae*, Supressor of Sec One (Sso1p) and Sec9p (SNAP-25) are target membrane SNARE proteins that bind synaptobrevin homolog 1 (Snc1p) to bring the target and vesicle membranes closer. Sec4p is a vesicle membrane, Ras-related, GTPase that binds the exocyst. EXOC7 and EXO1C bind the target membrane by the positively charged residues of phosphatidylinositol 4,5-bisphosphate (PI(4,5)P2) (shown as blue triangles with a white +). Adapted from^44^. The process results in tethering, docking and membrane fusion, releasing cargo contents. (**b**) In the absence of even one exocyst component, the rest of the components do not function properly, impairing vesicle tethering. This impairment inhibits vesicle docking and, thus, target and membrane fusion events do hot happen, so the vesicle contents remain in the untethered vesicle.

The third set of analyses focused on one exocyst component (EXOC7-H4-1) that was not expressed at any time point within the syncytium during the defense response or in control cells. However, EXOC7-H4-1 expression was confirmed to be increased in all 9 of the defense MAPK-OE lines by qRT-PCR (Fig. 9). These results indicate that aspects of plant secretion important for the defense process may occur outside of the parasitized root cells or their progenitors. This observation may explain why higher levels of suppressed *H. glycines* parasitism were not seen in prior experiments focusing on syncytium-expressing genes. Alternatively, overexpression of defense MAPKs may synthetically induce the expression of EXOC7-H4-1. In this case, EXOC7-H4-1 may or may not function in defense at all. To determine whether EXOC7-H4-1 functions in defense, it was cloned and used in overexpression and RNAi experiments. Transgenic EXOC7-H4-1-OE and EXOC7-H4-1-RNAi lines were assessed by qRT-PCR analyses with RPS21 used as a control, confirming their expected expression (Fig. 10). Analysis of the effect of altered EXOC7-H4-1 transgene cassette expression on root mass was performed, which demonstrated that the overexpression of EXOC7-H4-1 and RNAi cassette expression did not affect root mass (*p* > 0.05) (Fig. 11). In replicated functional analyses employing the same controls used in the previous study, EXOC7-H4-1-OE lines showed *H. glycines* parasitism that was significantly decreased by 58.8% in the wr and 58.3% pg of root system, as shown by the FI (*p* value < 0.001) (Fig. 12). In contrast, *H. glycines* parasitism in the EXOC7-H4-1-RNAi lines was significantly increased by 3.23-fold in the wr and 4.21-fold pg of root system, as shown by the FI (*p* value < 0.001) (Fig. 13).

## Discussion

An analysis of *G. max* exocyst components is presented here. This study examined whether exocyst components play a role during the defense response of *G. max* to the parasitic nematode *H. glycines*. The analysis began by the identification *G. max* exocyst genes from the most recent Wm82.a2.v1 genome. Then, exocyst genes expressed within the pericycle and surrounding cells prior to *H. glycines* infection (0 dpi) were determined. Follow-up studies then determined which exocyst genes are expressed within the syncytium during the defense response to *H. glycines* infection. The first of the time points selected for analysis was 3 dpi, at which point several cytological features did not differ between the susceptible and resistant cultivars. The second of the time points selected for analysis was 6 dpi, at which point the cytological features between the susceptible and resistant cultivars differed, characterizing each reaction. Complimentary analyses were conducted to identify whether the expression of any of the exocyst genes is under regulation by MAPKs. This analysis is undertaken because studies have demonstrated the importance of MAPKs to the defense response of *G. max* against *H. glycines*^50^. These genes are components of both ETI and PTI^50^. The subsequent functional, transgenic studies presented here demonstrated that exocyst genes function in defense. The experiments also identified an exocyst gene that is not expressed within the syncytium during the process of defense but functions in defense. Consequently, the experiments indicated that there processes important to defense occur both locally within the syncytium and outside of the syncytium.

This study began with the identification of all *G. max* exocyst genes through BLAST searches using the default parameters in Phytozome with *A. thaliana* exocyst protein sequences used as queries^72^. The analysis resulted in the identification of 61 genes that span the 8 exocyst gene families. These results are consistent with the composition of the exocyst in all eukaryotes, including plants^38,40–44,73^. The *G. max* exocyst genes include 5 EXOC1 genes, 2 EXOC2 genes, 5 EXOC3 genes, EXOC4 genes, 2 EXOC5 genes, 6 EXOC6 genes, 31 EXOC7 genes and 8 EXOC8 genes. Consequently, each gene family contains multiple gene copies, which is consistent with the duplicated nature of the *G. max* genome^74^. An analysis of the nature of these gene duplication events is beyond the scope of this study. However, Cvrčková et al*.*^73^ performed phylogenetic analyses of 10 different plant exocyst gene families and obtained important insights into the plant exocyst. The results showed that the small EXOC1, EXOC2, EXOC3, EXOC4 and EXOC5 gene families were likely amplified independently, late in the diversification of each plant lineage^73^. Furthermore, the small EXOC6 and EXOC8 gene families were likely amplified from a single ancestral gene^73^. In contrast, the very large EXOC7 gene family likely arose from early amplification of an ancestral gene in a common ancestor of land plants^73^. In each case, gene amplification leads to the diversification of paralog functions, which require further study. Therefore, the *G. max* EXOC7 gene family is notably expansive and consists of 31 members. The large size of the *G. max* EXOC7 gene family is consistent with observations in other land plants^73^.

*G. max* exocyst proteins show homology to those in *A. thaliana* ranging from a low identity of 42.61% (EXOC7-E2-2) to a high identity of 89.15% (EXOC3-5). However, two outliers in the EXOC8 gene family show a low identity of 60.96% (EXOC8-4) to a high identity of 75.30% (EXOC8-3). These outliers are EXOC8-6 (25.69% identity) and EXOC8-7 (25.66% identity). It is possible that these proteins are not EXOC8 homologs, and although they were included here, further study is required.

The main objective of this study was to understand whether the *G. max* exocyst plays a role in defense in the root. Toward that goal, exocyst gene accessions from the most recent *G. max* genome assembly (Wm82.a2.v1) were used for the identification of exocyst genes with Affymetrix GeneChip Soybean Genome Array probe set identifiers. This analysis was used to identify whether any of the exocyst genes exhibit expression specifically within the *H. glycines*-induced syncytium during the defense process. To the best of our knowledge, this is the only study to sue single-cell transcriptomic analyses from a multicellular organism to specifically identify exocyst genes. The analyses resulted in the identification of 27 of the 61 exocyst genes (44.26%) with an Affymetrix GeneChip Soybean Genome Array probe set identifier. Consequently, it was possible to identify whether any exocyst genes are expressed in the syncytium during the process of defense.

Within that list of 27 exocyst genes, 4 were expressed in the control population of root cells (pericycle and surrounding cells) that were sampled prior to *H. glycines* infection (0 dpi). After *H. glycines* infection, exocyst transcriptomic measurements were performed to detect the expression of 8 genes at 3 dpi during the defense response. Furthermore, 16 exocyst genes were identified as expressed at the 6-dpi time point during the defense response. The results showed an increase in the number of exocyst genes expressed during the course of *H. glycines* parasitism. This expression occurred specifically in *G. max* root cells during defense reactions (syncytium). Increases in the expression of all defense genes comprising a large gene family during the course of the defense response were seen in studies of the *G. max*-*H. glycines* pathosystem^34^. This study^34^ examined a family of 22 β-glucosidases predicted to have signal peptides and identified the secreted PEN2 homolog α-hydroxynitrile glucosidase (βg-4), which functions in *G. max* during its defense response against *H. glycines*^34^.

The results presented here indicate that the relative transcript abundance of these exocyst components may increase as a consequence of the *G. max* defense response. Furthermore, diverse cellular processes involving the *G. max* exocyst appear to lead to a successful defense response. For example, two different EXOC6 genes (EXOC6-1, EXOC6-6) and 8 different EXOC7 genes (EXOC7-A1-3, EXOC7-B1-2, EXOC7-D1-1, EXOC7-D1-2, EXOC7-E1-1, EXOC7-F1-1, EXOC7-G1-4 and EXOC7-H7-1) were seen here to function in the defense process (please refer to “Functional analysis of syncytium-expressing exocyst genes demonstrates a defense role” section: functional analysis of syncytium-expressed exocyst genes). The results indicated that different types of vesicles containing different cargos may function at specific times or in specific ways during the defense process.

Prior analyses have demonstrated the importance of the MAPK signaling platform to the *G. max* defense process against *H. glycines*^50^. MAPKs are part of a central, three-tier signal transduction platform shared by all eukaryotes. MAPKs permit cells to transduce signals into meaningful output for a variety of physiological and developmental purposes^75–77^. Recently, an analysis of the entire 32-member *G. max* MAPK gene family as it relates to defense against *H. glycines* was conducted^50^. The analysis led to the identification of 9 MAPKs that function in defense^50^: MAPK2, MAPK3-1, MAPK3-2, MAPK4-1, MAPK5-3, MAPK6-2, MAPK13-1, MAPK16-4 and MAPK20-250. In plants, most of the defense processes relating to MAPKs have been shown to involve MAPK3 and MAPK6. Until the work of McNeece et al*.*^50^, very little evidence regarding the possible defense roles of many of the other MAPKs was available. The observation that *G. max* MAPKs regulate the expression of exocyst components that function in defense argues strongly for the broad importance of MAPK signaling in the defense process against *H. glycines* parasitism.

The examination of syncytium-expressing exocyst genes shown here revealed that a number of these genes were differentially expressed in 1 or more transgenic MAPK lines. For example, EXOC7-B1-1 (an Exo70B1 homolog) had a lower relative transcript abundance in the MAPK2-RNAi line, in which the *G. max* defense response against *H. glycines* was suppressed^50^. These results are consistent with observations made in *A. thaliana* showing that some pathogen effectors impair Exo70B1 protein function through ubiquitination, leading to ETS^52^. In contrast, the relative transcript abundance of EXOC7-D1-1 was shown here to be higher in the MAPK3-2-OE line than in the corresponding control line. Furthermore, EXOC7-H7-1, which was expressed in the syncytium at the 3-dpi and 6-dpi time points, showed higher relative transcript abundance in all of the defense MAPK-OE lines (denoted MAPK-all-OE). The similar expression profile of EXOC7-H4-1 was shown here through its examination in functional experiments. However, EXOC7-H4-1 was not expressed in syncytial cells analyzed in the Affymetrix DCM study^31^. These results indicate that secretion processes outside of the vicinity of the syncytium are important for defense. If signaling processes outside of the syncytium function in defense, systemic processes such as systemic acquired resistance (SAR) may occur during the *G. max* defense process against *H. glycines*. *G. max* genes known to function in SAR, including the transcription factor NONEXPRESSOR of PR1 (NPR1), the lipase ENHANCED DISEASE SUSCEPTIBILITY 1 (EDS1) and the coiled-coil nucleotide-binding leucine rich repeat (CC-NB-LRR) resistance (R) protein NONRACE SPECIFIC DISEASE RESISTANCE1 (NDR1), have all been shown to function during the defense against *H. glycines* parasitism^33,50,78–84^. The observation that NDR1 functions in the *G. max* defense process against *H. glycines* is particularly noteworthy since NDR1 is required for ETI. In *A. thaliana*, NDR1 binds three R proteins: the CC-NB-LRR protein RESISTANCE TO PSEUDOMONAS SYRINGAE PV MACULICOLA1 (RPM1); RPM1-interacting 4 (RIN4); and the NB adaptor shared by APAF-1, certain *R* gene products and the CED-4 (ARC)-LRR (NB-ARC-LRR) gene RESISTANT TO P. SYRINGAE 2 (RPS2)^79,80,82,85–92^. NDR1 induces MAPK gene expression in the *G. max*-*H. glycines* pathosystem^50^.

Exocyst proteins functioning upstream of vesicle docking act to deliver callose to infection sites formed by pathogens^18,19^. This docking process employs the 20 S particle, which incorporates syntaxin (SYP)-containing SNARE. In *A. thaliana*, SYP121 and callose are delivered to defense sites in plants during resistance to *Botrytis graminis* f. sp. *hordei* by the ADP ribosylation factor (ARF)-GTP exchange factor GNOM^93^. Consistent with those observations, our prior analyses showed that overexpression of *G. max* syntaxin 121 resulted in increased callose deposition surrounding the *H. glycines* syncytium during the defense response^34^. In contrast, RNAi of *G. max* syntaxin 121 decreased callose deposition^34^. Subsequent follow-up studies identified the expression of 4 different CS genes in the syncytium during the defense process against *H. glycines*^51^. Furthermore, overexpression of the different CS genes resulted in a decrease in *H. glycines* parasitism, while RNAi of those same genes increased nematode parasitism^51^. These results provide evidence of processes requiring the exocyst to function in cells that are beyond the boundary of the syncytium during the defense response. *A. thaliana* Exo70H4 plays a role in callose deposition in trichomes, consistent with our observations and hypothesis^94^. The synthetic defense processes due to defense MAPK overexpression, as it relates to EXOC7-H4-1, are also under examination. Both qRT-PCR and RNA-seq analyses of controls that did not overexpress defense MAPKs detected EXOC7-H4-1 expression. These results provide evidence that EXOC7-H4-1 expression occurs at least in uninfected tissues that lack syncytia during the defense process.

In contrast to these results, the expression of a number of exocyst genes could not be analyzed by DCM because probe sets for these genes were not included in the Affymetrix GeneChip Soybean Genome Array. However, analysis of their expression was carried out through the use of data made available by a transgenic MAPK RNA-seq study^68^. The analysis identified a number of exocysts of genes that were differentially expressed in one or more transgenic MAPK-OE lines. For example, the EXOC7-E2-1 relative transcript abundance was higher in 8 of the 9 transgenic defense MAPK-OE lines. These *G. max* exocyst genes, however, were beyond the scope of the functional analysis presented here because their syncytium expression was not seen (M or NM) by the analytical methods used, referred to as n/a. These genes will be the focus of future analyses of the *G. max* exocyst.

Infection of genetically engineered exocyst-OE lines with *H. glycines* resulted in a 58–68% decrease in cysts per wr system and a 50–64% decrease in cysts pg of root system, depending on the exocyst component under study. Consequently, the *G. max* overexpression lines exhibited higher susceptibility to *H. glycines*. In contrast, RNAi studies revealed an increase in *H. glycines* parasitism of 3.16–3.77-fold in the wr system and a 4.13–5.68-fold increase in the cysts pg of root system when compared to those in the control. Consequently, the RNAi lines showed an increased susceptibility to *H. glycines*. These results are consistent with observations that syncytium-expressing genes function in the process of defense^32–34,37,50,51,83,84^. Furthermore, these results are consistent with observations showing that the vesicle transport apparatus that functions in vesicle docking and membrane fusion also functions in defense^32–34,37,50,51,83,84^.

Many of the genes under study in relation to *G. max* defense against *H. glycines* showed expression within the syncytium. However, the experiments did not rule out whether gene expression outside of the syncytium is important to the defense process. A recent study examining the entire *G. max* MAPK gene family demonstrated that 9 out of 9 MAPKs lacking expression within the syncytium have no role in defense^50^. These results argue against the functions of non-syncytium-expressed genes in defense. In contrast, 7 out of 12 syncytium-expressed MAPKs function in defense^50^. These results demonstrate that most of the MAPKs that function in defense are expressed within the syncytium. Affymetrix probe sets for the two other MAPKs that function in defense were lacking from the GeneChip Soybean Genome Array, so the syncytium expression of these MAPKs could not be determined^50^. Results demonstrating a defense role for genes expressed outside of the vicinity of the syncytium have yet to be determined. The results presented here reveal that the expression of certain exocyst genes (EXOC7-H4-1) occurs outside of the boundary of the parasitized root cells. In *A. thaliana*, the vesicle transport machinery involving the exocyst acts at some level to facilitate callose deposition^18,19,52,85,95–102^. Systemic processes outside of the vicinity of infection as well as structural modifications of *G. max* roots, including callose deposition and cell wall modification, have been observed^33,34,51,103^. These results are consistent with the observation that these components are coregulated and/or part of a feedback loop that further facilitates the expression of genes that function in the defense process^33,34^.

Over the past few years, a model of how the process of defense occurs in *G. max* as it reacts to *H. glycines* parasitism including the vesicle transport apparatus has been proposed^31–34,37,50,51^. This model has its origins in the demonstration that the 20 S component α-SNAP is specifically expressed within the syncytium during the defense process^31^. A number of studies have expanded on this theme, including analyses of the α-SNAP-binding protein syntaxin 31 and the other 20 S particle components, including SNAP-25, synaptobrevin, synaptotagmin, NSF and MUNC18^34^. These studies demonstrate the importance of the docking and membrane fusion steps in defense^34^. These vesicle transport steps are preceded by a tethering process performed by thee exocyst that is essential for membrane fusion to occur (Fig. 12). Early analyses indicated the importance of vesicle tethering during the *G. max* defense response to *H. glycines*^37^. Furthermore, and earlier analysis demonstrated that *G. max* Sec4, a protein that is known to function in tethering by binding EXOC6, plays a defensive role against *H. glycines* parasitism^37^. Related experiments have demonstrated that the mechanism by which vesicles are delivered to the cell periphery, which functions through myosin XI, is also important during defense^51^. These results all point toward the function of the exocyst at a crucial point in the delivery of vesicles to the site of membrane fusion and plant secretion in the defense process of *G. max* against *H. glycines* parasitism. Furthermore, it appears possible that specific exocyst genes, which are likely the products of duplication events, may function in specific ways to increase the breadth of the defense response or general health of the plant^73^. With regard to root-organism interactions, the conserved nature of the exocyst indicates that its function is not limited to the *G. max*-*H. glycines* pathosystem, indicating the broad importance of this study.

## Methods

### Candidate gene selection

The Phytozome portal (https://phytozome.jgi.doe.gov) houses the *G. max* genome sequence and information about its assembly and annotation, making acquisition of the protein sequences of the entire exocyst gene family possible^72^. *G. max* exocyst protein accessions were identified based on comparisons to *A. thaliana* protein sequences in Phytozome using the Basic Local Alignment Search Tool (BLAST) with the default settings^72^. These default settings were as follows: target type: proteome; program: BLASTP (protein query to protein database); expect (E) threshold: − 1; comparison matrix: BLOSUM62; word (W) length: default = 3; number of alignments to show: 100 allowing for gaps and filter query.

Identification and selection of the *G. max* exocyst defense genes for use in functional transgenic studies were carried out by using the gene expression data from Matsye et al*.*^31^. These data were obtained through microarray analyses using the GeneChip Soybean Genome Array (Affymetrix). In the study, Matsye et al*.*^31^ infected two different *G. max* cultivars that are susceptible or resistant to the *H. glycines* cultivar under study. *G. max*_[Peking/PI 548402]_ and *G. max*_[PI 88788]_ can be infected with *H. glycines*_[race 14/HG-type 1.3.6.7/TN8]_, rendering them susceptible. In contrast, *G. max*_[Peking/PI 548402]_ and *G. max*_[PI 88788]_ can be infected with *H. glycines*_[NL1-Rhg/HG-type 7/race 3]_, rendering them resistant. Laser microdissection (LM) was used to collect pericycle (control) cells prior to *H. glycines* infection (0 days postinfection [dpi] control). Syncytia during the resistance process were collected at 3 and 6 dpi. Complementary DNA (cDNA) probes were generated from RNA samples collected at 0, 3 and 6 dpi. The resulting cDNA probes were used in hybridization experiments with Affymetrix GeneChip Soybean Genome Arrays^31,104^. These arrays are composed, in part, of 37,744 *G. max* probe sets covering 35,611 transcripts^31,104^. The experiments were run in triplicate^31,104^. This experimental process resulted in the production of 6 total arrays for each time point (*G. max*_[Peking/PI 548402]_: arrays 1–3; *G. max*_[PI 88788]_: arrays 1–3), which were used to determine whether the presence of transcript corresponding to the probe set was demonstrably different from zero (present [P]), uncertain (marginal [G]), or not demonstrably different from zero (absent [A])^31,104^. For our purposes, a gene was considered to be measured [M] when the probe signal was detectable above a threshold (*p* < 0.05) on all 6 arrays (the 3 arrays each from *G. max*_[Peking/PI 548402]_ and *G. max*_[PI 88788]_)^31,104^. For the analysis presented here, the expression of an exocyst gene was considered not measured (NM) if the probe signal was not detected at a statistically significant level (*p* ≥ 0.05) on any of the 6 arrays. For some genes, no corresponding probe set was fabricated onto the microarray. In these cases, gene expression was not determined and considered not applicable (n/a). For this part of the analysis, the Affymetrix annotations were mapped to the original *G. max* genome release (Wm82*.*a1*.*v1*.*1) since only that annotation was available at the time of the analysis^31^. Here, these older annotations are compared to the updated, most recent *G. max* Wm82.a2.v1 genome assembly and annotation.

### RNA sequencing

RNA sequencing (RNA-seq) data were obtained from the experiments of McNeece et al*.*^50^ and Alshehri et al*.*^68^. These studies examined the *G. max* MAPK gene family as it relates to the defense of *G. max* against *H. glycines* parasitism and showed that 9 of the 32 *G. max* MAPKs function during the defense reaction against *H. glycines* parasitism, naming these MAPKs defense MAPKs^50^. Single replicate RNA-seq experiments examining the 9 defense MAPKs were performed using RNA isolated from transgenic lines in which the targeted MAPK genes were either overexpressed (OE) or inhibited via RNAi^50,68^. The defense MAPKs examined were MAPK2 (Glyma.06G029700), MAPK3-1 (Glyma.U021800), MAPK 3-2 (Glyma.12G073000), MAPK 4-1 (Glyma.07G066800), MAPK 5-3 (Glyma.08G017400), MAPK6-2 (Glyma.02G138800), MAPK 13-1 (Glyma.12G073700), MAPK16-4 (Glyma07g38510) and MAPK20-2 (Glyma.14G028100), and pRAP15-*ccd*B and pRAP17-*ccd*B served as corresponding controls^50,68^. RNA was isolated from the 9 defense MAPK-OE and MAPK-RNAi lines and their respective controls, and the RNA sequences were deposited and made publicly available^68^. For the experimental purposes presented here, an additional goal was the identification of exocyst genes whose expression was induced or suppressed by the different studied MAPKs. Expression of the exocyst genes that met the differential expression criterion in the RNA-seq experiments (± 1.5-fold change in expression, *p* < 0.05) was confirmed by quantitative real-time PCR (qRT-PCR) as described in the Materials and Methods section (“cDNA synthesis” and “qRT-PCR assessment of gene expression” sections). Expression of the remaining exocyst genes that did not meet the differential expression criterion in the RNA-seq analyses (NDE) was not confirmed by qRT-PCR. The *G. max* genome accessions were used to mine exocyst RNA-seq gene expression data from the defense MAPK RNA-seq study and are shown in the analysis^50,68^. The *G. max* accession numbers whose RNA-seq data are presented were derived from the most recent *Glycine max* Wm82.a2.v1 annotation. The exocyst accession numbers were further manually confirmed with Phytozome to confirm their accuracy (as of February 15, 2020)^72,74^.

### Functional testing of candidate defense genes

The candidate *G. max* exocyst defense gene sequences were extracted from Phytozome^72,74^, cloned and overexpressed in the *H. glycines*-susceptible cultivar *G. max*_[Williams 82/PI 518671]_^50^. In addition, the candidate exocyst defense genes were cloned for RNAi in the *H. glycines*-resistant cultivar *G. max*_[Peking/PI 548402]_^50^. PCR primer sequences were developed against the exocyst component transcript sequences from the recent *Glycine max* Wm82.a2.v1 annotation and confirmed to match the Wm82.a2.v1 genome (February 15, 2020) (Supplementary Table S3). Candidate *G. max* exocyst defense gene amplicons were synthesized by PCR using the AccuPrime Taq Polymerase System (Invitrogen) according to the manufacturer’s instructions with an Eppendorf AG Mastercycler Pro S model 6,325 PCR gradient PCR thermocycler. The reaction conditions were dependent on the nucleotide composition of the amplicon and PCR primer. In general, DNA melting was carried out at 95 °C for 2 min, followed by another 30-s melt at 95 °C. Primer annealing conditions were empirically determined through gradient PCR for 30 s. Primer extension was carried out at 68 °C for 1 min per 1,000 base pairs of the sequence. This process was carried out for 35 cycles, followed by a final step at 68 °C for 10 min, with the reaction completed at 4 °C. The PCR product was run on a 1% agarose gel. The amplicons were removed from the gel and purified using the Wizard SV Gel and PCR Clean-Up System (Promega) according to the manufacturer’s instructions. Subsequently, the amplicon was ligated into the pENTR/D-TOPO entry vector using the pENTR/D-TOPO Cloning Kit (Invitrogen) according to the manufacturer’s instructions. The reaction contents were transformed into One Shot TOP10 chemically competent *E. coli* (TOP 10) (Invitrogen) cells according to the manufacturer’s instructions as described. Cells were selected on Luria–Bertani (LB) agar plates containing 50 μg/ml kanamycin. Plasmid DNA was isolated from selected colonies using the Wizard *Plus* SV Minipreps DNA Purification System (Promega) according to the manufacturer’s instructions. The DNA sequences were confirmed by Sanger sequencing. Subsequently, the exocyst amplicons were ligated to Gateway-compatible overexpression (pRAP15) or RNAi (pRAP17) destination vectors using Gateway LR Clonase Enzyme Mix (Invitrogen) according to their instructions to transfer the candidate *G. max* exocyst resistance gene amplicon into the respective destination vectors. Nonengineered pRAP15 and pRAP17 vectors served as experimental controls; these vectors contained the *ccd*B gene where the candidate *G. max* exocyst defense gene amplicon would otherwise be following directional insertion during the LR clonase reaction. Based on this feature, the nonengineered pRAP15-*ccd*B (overexpression control) and pRAP17-*ccd*B (RNAi control) vectors were suitable controls to account for any nonspecific effects of gene overexpression or RNAi^50^. The reaction contents were transformed into chemically competent *E. coli* TOP 10 cells according to the manufacturer’s instructions as described. Cells were selected on LB agar plates containing 5 μg/ml tetracycline. *E. coli* colonies containing the gene of interest (GOI) after transformation with pRAP15/17-GOI were grown in 3 ml of liquid LB medium and chemically selected with 5 μg/ml tetracycline overnight at 37 °C. Plasmid preps (Promega) of these liquid cultures were carried out according to the manufacturer’s instructions. Gene-specific primers were used to confirm the presence of each exocyst gene (Supplementary Table S3). The pRAP15/17 destination vectors confirmed to have the exocyst gene amplicon were transformed into chemically competent *Agrobacterium rhizogenes* K599 (K599)^50^ via freeze–thaw transformation^50^. In this procedure, 250 μl of K599 cells was thawed on ice. A sufficient amount of plasmid DNA (0.1–1 μg) was added to K599 cells and gently mixed. The mixture of K599 cells and plasmid DNA was incubated on ice for 5 min and then subsequently transferred to liquid N_2_ for 5 min. The mixture was transferred to a 37 °C water bath for 5 min. The contents were then transferred to a culture tube with 1 ml of LB medium, placed in a shaking incubator, and incubated at 28 °C for 2 h. The cells were then collected by centrifugation for 2 min at 5,000 rpm, resuspended in 200 μl of LB medium and spread on LB agar plates containing 5 μg/ml tetracycline for chemical selection at 28 °C. After 2 days, K599 colonies that underwent genetic transformation were picked for confirmation of the DNA cassette with *G. max* exocyst gene-specific primers. Colonies harboring the appropriate plasmid were then grown in 250 ml of LB medium containing 5 μg/ml tetracycline at 28 °C in a shaking incubator^50^.

### Production of transgenic plants for functional experiments

A solution of K599 cells transformed with the appropriate vector construct was pelleted by centrifugation in a Sorvall RC6 Plus Superspeed Centrifuge at 4 °C for 20 min. The resulting pellet of K599 cells was resuspended in Murashige and Skoog medium containing vitamins (MS) (Duchefa Biochemie) and 3.0% sucrose at pH 5.7 (MS medium)^105^. Transgenic *G. max* production began when the root of each 1-week-old plant was cut off at the hypocotyl with a new, sterile razor blade that had been immersed in the K599 cell solution in a Petri dish. This procedure allowed the transformed K599 cells access to the wound made by removal of the root. Subsequently, 25 root-less plants were placed in a 140-ml glass beaker with 25 ml of transformed K599 cells in MS medium. The plants were placed under vacuum using the VP60 Two Stage Vacuum Pump (CPS Products, Inc.) in a Bel-Art “Space Saver” polycarbonate vacuum desiccator with a clear polycarbonate bottom (Bel-Art) for 5 min. The plants were then left under vacuum for 10 min. The vacuum was then slowly released, allowing the transformed K599 cells to further enter the plant tissue. After cocultivation, the cut ends of *G. max* were individually placed 3–4 cm deep into fresh coarse grade A-3 vermiculite (Palmetto Vermiculite) in 50-cell propagation trays (725602C) held in standard flats (710245C) with holes in the bottom (T.O. Plastics). The plant trays are were placed in sterilize 25-qt./23-L modular latched boxes (Sterlite) that were then covered with their lids. The covered modular latched boxes were placed at a distance of 20 cm from standard fluorescent cool white 4,100-K/32-W bulbs emitting 2,800 lumens (Sylvania) for 5 days at ambient laboratory temperature (22 °C). The plants were transferred to the greenhouse and removed from the modular latched boxes for recovery for 1 week. Visual selection of transgenic *G. max* roots was carried out with the enhanced green fluorescent protein (eGFP) reporter using a Dark Reader Spot Lamp (SL10S) (Clare Chemical Research)^50^. Roots exhibiting eGFP reporter expression also possessed the candidate defense gene expression cassette, and each had its own promoter and terminator sequences. Gene transfer occurred because K599 cells transported the DNA cassettes between the left and right borders of the pRAP15 and pRAP17 destination vectors into the root cell chromosomal DNA. The result was a stable transformation event in the root somatic cell, even though the DNA cassette had not been incorporated into the germline. Roots subsequently developed from the transgenic cell over a period of a few weeks. The resultant genetically mosaic plants had a nontransgenic shoot with a transgenic root system. Therefore, in the experiments presented here, each individual transgenic root system is an independent transformant line. The transgenic plants were each planted in a Ray Leach “Cone-tainer” (SC10) (Stuewe and Sons, Inc.) secured in a Ray Leach Tray (RL98) (Stuewe and Sons, Inc.) in sandy (93.00% sand, 5.75% silt, and 1.25% clay) soil and allowed to recover for two weeks prior to the start of the experiment^50^. The functionality of the genetic constructs in *G. max* was confirmed by qRT-PCR (please refer to the quantitative real-time PCR [qRT-PCR]-related Sects. 4.5 and 4.6).

### cDNA synthesis

*G. max* root RNA was isolated using the UltraClean Plant RNA Isolation Kit (Mo Bio Laboratories, Inc.) according to the manufacturer’s instructions^50^. The RNA was quantified using a NanoDrop 2000 spectrophotometer (Thermo Fisher Scientific) according to the manufacturer’s instructions. Genomic DNA was removed from the RNA with amplification-grade DNase I (Invitrogen) according to the manufacturer’s instructions. With oligo d(t) used as the primer, the SuperScript First Strand Synthesis System for RT-PCR (Invitrogen) was used to synthesize cDNA from mRNA according to the manufacturer’s instructions. Genomic DNA contamination was assessed using a cupin (Glyma.20G148300) primer pair that amplifies DNA across an intron (Supplementary Table S3)^32^. The PCR experiments yielded differently sized products based on the presence or absence of that intron^32^.

### qRT-PCR assessment of gene expression

Candidate exocyst defense gene expression in transgenic *G. max* was assessed by qRT-PCR using the StepOnePlus Real-Time PCR System (Applied Biosystems), TaqMan 6-carboxyfluorescein (6-FAM)-labeled probes and Black Hole Quencher-1 (BHQ1) (MWG Operon) (Supplementary Table S3) according to the manufacturer’s instructions^50^. A qRT-PCR control designed from a ribosomal S21 (RPS21) protein-coding gene (Glyma.15G147700) was used in the *G. max* experiments (Supplementary Table S3)^50^. Fold changes in gene expression caused by the genetic engineering events were calculated using the 2^−ΔΔ*CT*^ method^50,69^. Student’s *t*-test was used to calculate the *p* values for the replicate qRT-PCR experiments^70^. The procedures followed those presented by McNeece et al*.*^50^.

### Assaying the effect the genetic engineering events on nematode parasitism

Infection of the transgenic plants with *H. glycines* was performed according to the procedures described by Sharma et al*.*^34^. *H. glycines* eggs were obtained from cysts collected from 60-day-old, greenhouse-grown *G. max* stock cultures. The cultures were maintained in 500-cm^3^ polystyrene pots. Stock *H. glycines* cysts were purified by sucrose flotation^106^. *G. max* roots that contained *H. glycines* cysts were washed through nested sieves with pore sizes of 850 μm and 250 μm. The *H. glycines* cysts were collected from the 250-μm sieve after this procedure and ground with a mortar and pestle to release the eggs. The *H. glycines* eggs were obtained after gravitational sieving followed by sucrose centrifugation. The *H. glycines* eggs were recovered with a 75-μm sieve nested over a 25-μm sieve. *H. glycines* J2s were collected from hatched eggs in a modified Baermann funnel placed on a Slide Warmer (model 77) (Marshall Scientific) at 28 °C. *H. glycines* eggs hatched from days 4 to 7. *H. glycines* J2s were collected on a sieve with a 25-μm pore sire and placed in 1.5-ml tubes. The tube and its contents were centrifuged at 10,000 rpm for 1 min, washed with distilled sterile water and centrifuged again. The J2s were concentrated by centrifugation in an IEC clinical centrifuge for 30 s at 1,720 rpm to a final optimized concentration of 2,000 pi-J2/ml. Each root was inoculated with one ml of *H. glycines* at a concentration of 2,000 J2s/ml per root system (per plant). Infection was allowed to proceed for 30 days. At the end of the experiment, the cysts were collected over nested 20- and 100-mesh sieves^34^. Furthermore, the soil was washed several times, and the rinse water was sieved to assure collection of all cysts for enumeration of the female index (FI)^34^.

The FI, the community-accepted standard representation of the obtained data^24^, was calculated by a procedure originally described by Golden et al*.*^24^ as follows and employed for functional transgenic experiments^50^: FI = (Nx/Ns) × 100. In the procedure employed here, Nx was the pRAP-exocyst gene-transformed (experimental) line. Ns was the pRAP-*ccd*B (control) line^34^. The FI was calculated as the number of cysts per whole root (wr) system grown within 100 cc of soil and the number of cysts per gram (pg) of root system^50^. Historically, analysis by wr system has been the method of choice for data presentation^24^. Analysis of the number of cysts pg of root system, however, accounts for possible changes in root growth caused by the influence of the overexpression or RNAi of the candidate *G. max* exocyst defense gene. Three biological replicates consisting of 10–20 individual transgenic plants each were made for each construct. The results were statistically examined using the Mann–Whitney–Wilcoxon (MWW) rank-sum test, a nonparametric test of the null hypothesis that does not require the assumption of a normal distribution, with a cutoff of *p* < 0.05^50,71^.

## Supplementary information

Supplementary Table 1.

Supplementary Table 2.

Supplementary Table 3.

## Data Availability

Data relevant to the study is presented here as supplemental data.

## Abbreviations

DCM: Detection call methodology
wr: Whole root system
pg: Per gram
SAR: Systemic acquired resistance
